# Improving the In Vivo Stability of [^52^Mn]Mn(II) Complexes with 18-Membered Macrocyclic Chelators for PET Imaging

**DOI:** 10.1021/acs.jmedchem.4c00812

**Published:** 2024-06-27

**Authors:** Charlene Harriswangler, James M. Omweri, Shefali Saini, Laura Valencia, David Esteban-Gómez, Madalina Ranga, Nicol Guidolin, Zsolt Baranyai, Suzanne E. Lapi, Carlos Platas-Iglesias

**Affiliations:** † Universidade da Coruña, Centro Interdisciplinar de Química e Bioloxía (CICA) and Departamento de Química, Facultade de Ciencias, A Coruña 15071, Galicia, Spain; ‡ Department of Chemistry, University of Alabama at Birmingham, Birmingham, Alabama 35205, United States; § Department of Radiology, University of Alabama at Birmingham, Birmingham, Alabama 35294, United States; ∥ Departamento de Química Inorgánica, Facultad de Ciencias, Universidade de Vigo, As Lagoas, Marcosende 36310, Pontevedra, Spain; ⊥ Bracco Imaging SpA, CRB Trieste, AREA Science Park, ed. Q—S.S. 14 Km 163,5, 34149 Basovizza, TS, Italy

## Abstract

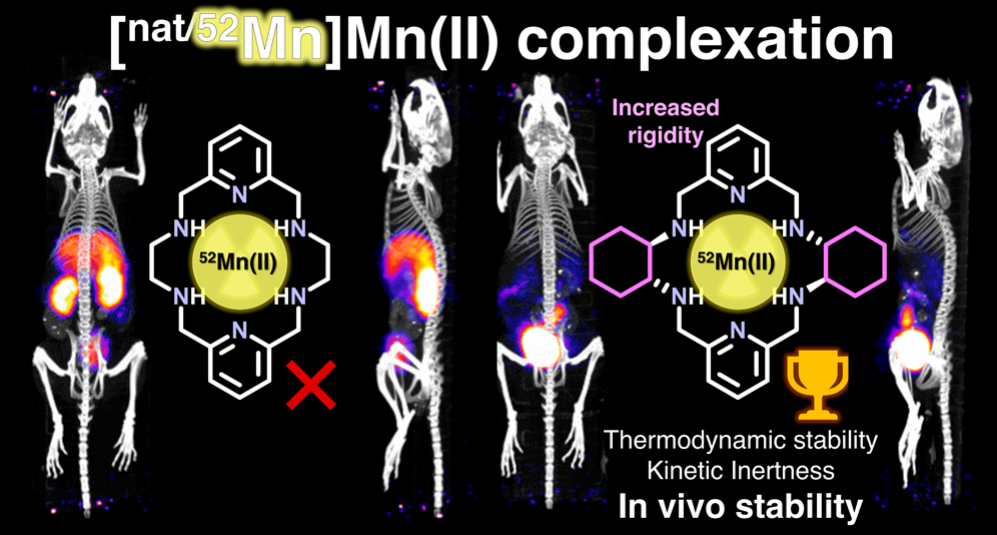

We report the [^nat^Mn/^52^Mn]Mn(II) complexes of the macrocyclic chelators PYAN [3,6,10,13-tetraaza-1,8(2,6)-dipyridinacyclotetradecaphane] and CHXPYAN [(4^1^*R*,4^2^*R*,10^1^*R*,10^2^*R*)-3,5,9,11-tetraaza-1,7(2,6)-dipyridina-4,10(1,2)-dicyclohexanacyclododecaphane]. The X-ray crystal structures of Mn-PYAN and Mn-CHXPYAN evidence distorted octahedral geometries through coordination of the nitrogen atoms of the macrocycles. Cyclic voltammetry studies evidence reversible processes due to the Mn(II)/Mn(III) pair, indicating that the complexes are resistant to oxidation. CHXPYAN forms a more thermodynamically stable and kinetically inert Mn(II) complex than PYAN. Radiochemical studies with the radioactive isotope manganese-52 (^52^Mn, *t*_1/2_ = 5.6 days) evidenced better radiochemical yields for CHXPYAN than for PYAN. Both [^52^Mn]Mn(II) complexes remained stable in mouse and human serum, so in vivo stability studies were carried out. Positron emission tomography/computed tomography scans and biodistribution assays indicated that [^52^Mn]Mn-PYAN has a distribution pattern similar to that of [^52^Mn]MnCl_2_, showing persistent radioactivity accumulation in the kidneys. Conversely, [^52^Mn]Mn-CHXPYAN remained stable in vivo, clearing quickly from the liver and kidneys.

## Introduction

Harnessing the properties of the metallic elements in vivo for medicinal applications most often requires the input of the field of coordination chemistry.^1−3^ The formation of coordination compounds allows safe delivery of the metallic element into the body, avoiding negative side effects related to the release of metal. Coordination compounds are used for various biomedical applications, including both therapy and imaging.^4,5^ Medical imaging requires the use of metal complexes for different techniques, such as the contrast agents used in magnetic resonance imaging (MRI),^6^ which are most often coordination compounds based on gadolinium(III). There are also many different metals that are used as radiotracers for positron emission tomography (PET), with the most common metallic isotopes being gallium-68 (^68^Ga, *t*_1/2_ = 67.7 min), copper-64 (^64^Cu, *t*_1/2_ = 12.7 h), and zirconium-89 (^89^Zr, *t*_1/2_ = 78.4 h).^7^

Manganese is a promising metal for both MRI and PET applications. It has been proposed as an alternative to MRI contrast agents based on gadolinium due to the endogenous character of the Mn(II) ion.^8^ The Mn(II) complexes used for this application must be coordinatively unsaturated, so that at least one exchanging water molecule can bind to the metal ion.^9,10^ This can, at times, make the search for a stable Mn(II) complex more difficult, as one may need to sacrifice additional donor arms so that the water molecule can bind, with some of the most stable Mn(II) complexes having coordination number (CN) 8.^11,12^ In the case of PET radiotracers based on Mn(II) isotopes, this condition is no longer in place as no water exchange is necessary for the acquisition of PET images. This allows the broadening of the library of potential chelators for Mn(II) complexation with interest in radiopharmaceutical applications. Additionally, the amount of compound necessary for the acquisition of PET images is much lower than that required for the use of contrast agents in MRI.^13^

One of the proposed manganese radioisotopes for PET imaging is manganese-52 (^52^Mn, *t*_1/2_ = 5.6 days), a cyclotron-produced, long-lived positron emitter that can be used to obtain high-resolution images several days after injection.^13−15^ This can allow for longer studies of biological processes than those that are carried out with traditional PET radioisotopes and also pairs nicely with the biological half-lives of some antibodies.^16−18^ Therefore, it is vital that the chosen chelating unit forms a highly inert complex with [^52^Mn]Mn(II), to ensure that the metal ion is not released in vivo before the images of interest are recorded.

The selection of an adequate chelator for [^52^Mn]Mn(II) must consider several different factors such as thermodynamic stability and kinetic inertness of the resulting complex. High-spin Mn(II) complexes do not have any ligand field stabilization energy (LFSE) and are generally kinetically labile and are less stable than other complexes of divalent first transition series metals.^11^ When designing a chelator, one of the aspects coordination chemists will consider is Pearson’s hard–soft acid–base principle.^19,20^ According to Pearson’s original classification, Mn(II) falls into the category of hard acids and will therefore pair best with hard bases containing oxygen and nitrogen donor atoms.^19^

Another interesting property of manganese complexes is the possibility of developing redox-responsive systems, taking advantage of the Mn(II)/Mn(III) pair.^21^ This strategy has been investigated for Mn-based MRI contrast agents, exploiting changes in ^1^H relaxation enhancement effects upon varying the oxidation state of the metal ion.^22,23^ Though application to radiochemistry could be more difficult, the development of the radiochemistry of redox-responsive Mn(II)-based pharmaceuticals could pose an interesting challenge.^21^ Therefore, studying the electrochemistry of Mn(II) complexes is an important assay to assess the properties of these systems.

**Figure 1:**
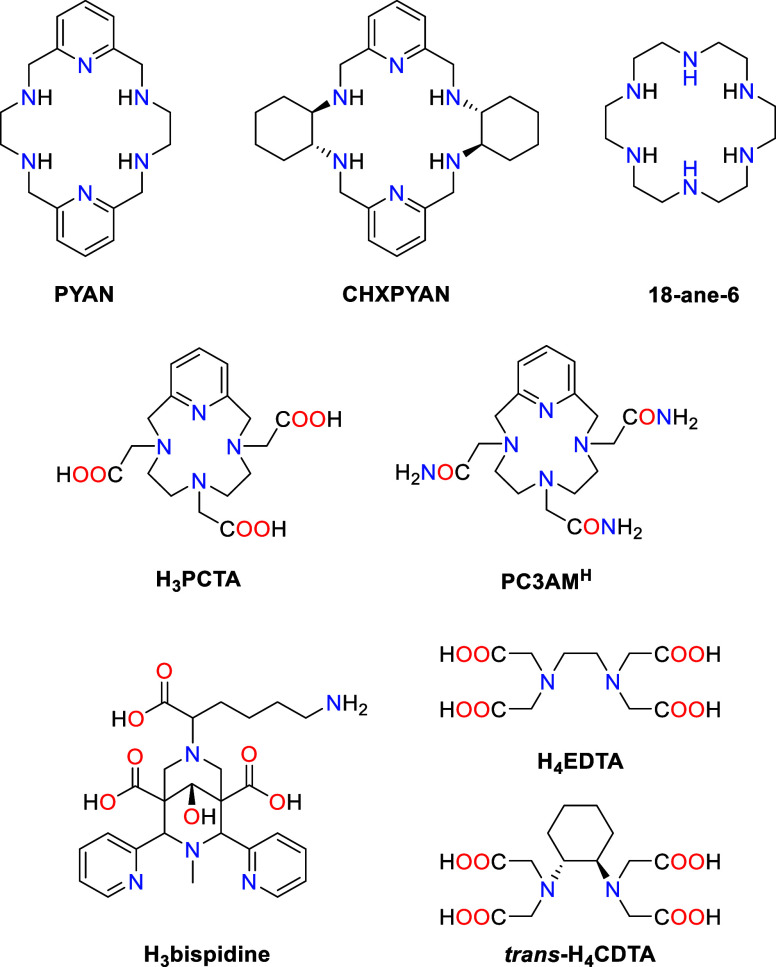
Chelators discussed in this work.

Herein, we provide a detailed investigation of the coordination of [^nat^Mn/^52^Mn]Mn(II) by 18-membered macrocycles PYAN and CHXPYAN (Figure 1). These systems were first described by Jackels et al. in the 1990s, including an investigation of the Mn(II) complexes of these chelators, although they were not proposed for biomedical applications.^24,25^ First, we report a study on the coordination chemistry with stable Mn(II), including X-ray crystallography, cyclic voltammetry, stability constant determination, and dissociation kinetics. Once these studies were complete, radiolabeling assays were carried out using [^52^Mn]Mn(II), and the stability of the complexes was studied through in vitro and in vivo investigations. Through these studies, it was determined that CHXPYAN is the superior chelator and is very promising for further investigation.

## Results and Discussion

### Synthesis and X-ray Structures

**Figure 2:**
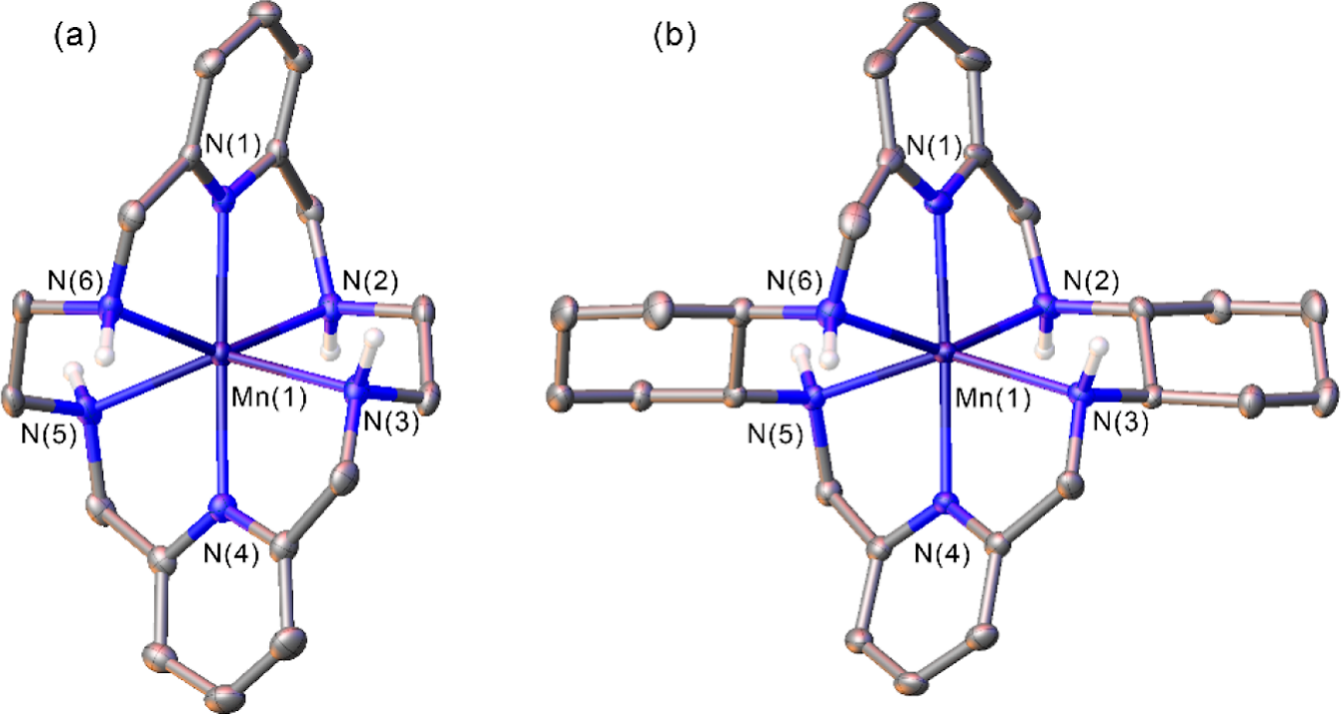
Views of the Mn-PYAN (a) and Mn-CHXPYAN (b) complexes present in crystals of [Mn(PYAN)](PF_6_)(NO_3_) and [Mn(CHXPYAN)](PF_6_)_2_. The ORTEP plots are at the 50% probability level (deposition numbers CCDC 2335672 and 2335674).

The preparation of ligands PYAN and CHXPYAN was carried out following previously reported procedures.^24,26^ While the Mn(II) complex of PYAN was previously reported as the bromide salt,^27^ to the best of our knowledge, through a search of the CCDC database, the CHXPYAN complex had not. Reaction of the ligands with Mn(NO_3_)_2_ in ethanol afforded the corresponding water-soluble Mn(II) complexes, with high-resolution mass spectra and high-performance liquid chromatography (HPLC) analysis confirming their formation (Figures S1–S4, Supporting Information). Crystals suitable for X-ray diffraction studies were obtained upon the addition of KPF_6_ to solutions of the complexes in water–acetonitrile. For Mn-PYAN, we obtained two different structures that show slightly different bond distances and angles of the metal coordination environments. Relevant bond distances and angles are compared with those reported previously^27^ for [Mn(PYAN)][MnBr_4_] in Table 1, while views of the structures of the complexes are presented in Figure 2.

**Table 1:** Interatomic Distances (Å) and Bond Angles (deg) of the Metal Coordination Environments in the Mn(II) Complex Crystal Structures

	[Mn(PYAN)](PF_6_)_0,5_(NO_3_)_1,5_	[Mn(PYAN)](PF_6_)_2_^a^	[Mn(PYAN)][MnBr_4_]^27^	[Mn(CHXPYAN)](PF_6_)_2_^b^
Mn(1)–N(1)	2.1992(11)	2.225(3)	2.194	2.170(3)
Mn(1)–N(2)	2.3004(11)	2.299(3)	2.286	2.262(3)
Mn(1)–N(3)	2.2823(12)	2.305(3)	2.302	2.288(3)
Mn(1)–N(4)	2.2044(12)	2.228(3)	2.197	2.173(3)
Mn(1)–N(5)	2.2948(12)	2.302(3)	2.277	2.284(3)
Mn(1)–N(6)	2.2897(11)	2.285(3)	2.274	2.330(3)
N(1)–Mn(1)–N(4)	171.70(4)	177.71(11)	167.01	176.84(12)
N(2)–Mn(1)–N(6)	146.60(4)	145.09(12)	149.22	148.32(11)
N(3)–Mn(1)–N(5)	146.28(4)	145.14(11)	147.45	146.91(10)

a The asymmetric unit contains three complex entities with slightly different bonds and angles.

b The asymmetric unit contains two complex entities with slightly different bonds and angles.

The Mn–N distances involving pyridyl N atoms are shorter than those involving amine N atoms by ∼0.1 Å, as usually observed for Mn(II) complexes.^28−32^ The distances to pyridyl N atoms [Mn(1)–N(1) and Mn(1)–N(4)] are significantly shorter in Mn-CHXPYAN than in Mn-PYAN, likely as a result of the high rigidity introduced by the cyclohexyl spacers. The presence of a long Mn–N distance in Mn-CHXPYAN [Mn(1)–N(6) = 2.330(3) Å] appears to be necessary to accommodate these two short distances in the metal coordination environment. The coordination polyhedra around the Mn(II) ions can be described as severely distorted octahedra, as evidenced by shape measurements (Table S1, Supporting Information).^33^ Indeed, the *trans* angle involving the pyridyl donor atoms [N(1)–Mn(1)–N(4)] is relatively close to the ideal value for an octahedron (180°), but the two remaining trans angles show large deviations from linearity (Table 1).

The two salts of the Mn-PYAN complex crystallize in the monoclinic space groups (*C*2/*c* and *P*2_1_/*c*), with crystals containing the centrosymmetrically related (δδ)/(λλ) enantiomeric pair. In this nomenclature, δ and λ identify the different helicities associated with the gauche conformations of the five-membered chelate rings that are formed upon coordination of the ethylenediamine units of the ligand.^34,35^ Interestingly, the previously described structure contains the (δλ)–(λδ) enantiomeric pair of the chelates. This indicates that the counterion influences the conformation adopted by the complex, which presents a certain degree of fluxionality.

The structure containing the Mn-CHXPYAN complex crystallizes in monoclinic space group *P*2_1_, displaying only the (λλ) conformation of the chelates. This is imposed by the use of the enantiomerically pure (1*R*,2*R*)-cyclohexane-1,2-diamine precursor. The Zn(II) complex of CHXPYAN prepared with racemic (±)*trans*-1,2-diaminocyclohexane was previously described by Bligh and co-workers.^36^ In this structure, which crystallizes in the centrosymmetric *P*2_1_/*c* monoclinic space group, the chelates adopt a λλ conformation in the case of the *RRRR* macrocycle (the same conformation that our system has) and a δδ conformation in the case of the *SSSS* isomer.

### Cyclic Voltammetry

**Figure 3:**
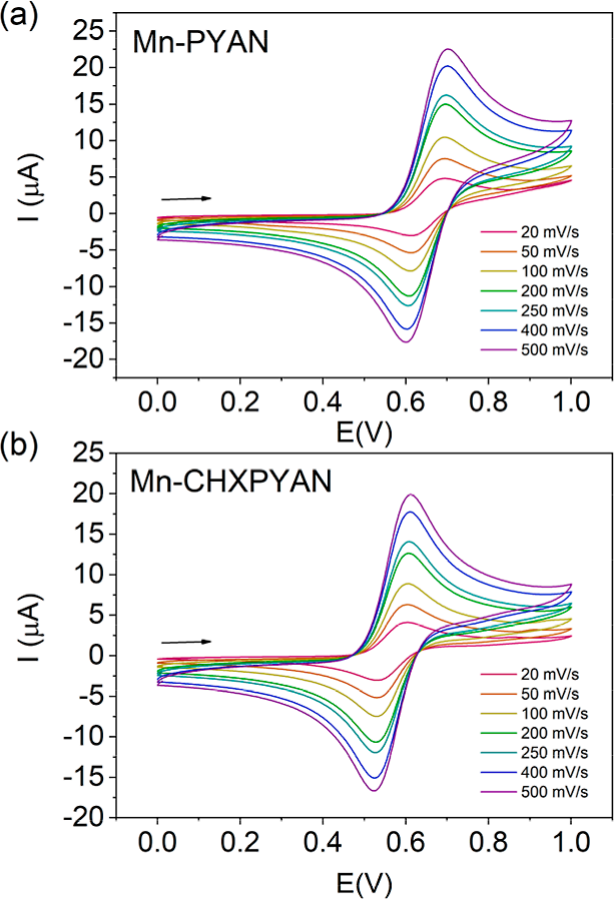
Cyclic voltammograms recorded for solutions (∼2 mM) of Mn-PYAN (a) and Mn-CHXPYAN (b) in 0.15 M NaCl referenced to the Ag/AgCl electrode. The arrow indicates the direction of the scan.

Cyclic voltammetry experiments were performed with Mn-PYAN and Mn-CHXPYAN, using aqueous solutions with a complex concentration of 2 mM, containing 0.15 M NaCl as the supporting electrolyte (pH ∼ 6.5). The cyclic voltammograms obtained (Figure 3) show well-defined anodic and cathodic waves due to the Mn(II)/Mn(III) pair, which are characteristic of reversible or quasi-reversible processes. At the lowest scan rate (20 mV/s), the peak-to-peak separation (Δ*E*_p_) is slightly smaller for Mn-CHXPYAN than for Mn-PYAN, with values of 73 and 80 mV, respectively. When increasing the scan rate, Δ*E*_p_ increases slightly, with values of 85 and 102 mV, respectively, at the highest scan rate (500 mV/s). This situation is characteristic of electrochemically quasi-reversible processes, although for Mn-CHXPYAN, the values of Δ*E*_p_ are lower and present less variation with the scan rate. Plots of peak current versus the square root of the scan rate (Figures S4 and S5) show linear dependency for both complexes, indicating that the electrochemical processes that take place are diffusion-controlled.

The half wave potentials (*E*_1/2_) determined for Mn-PYAN and Mn-CHXPYAN are 652 and 567 mV (vs Ag/AgCl), which indicates that Mn-PYAN is somewhat more resistant to oxidation than Mn-CHXPYAN. This is likely related to ligand field effects due to the more symmetrical octahedral coordination around the metal ion in Mn-CHXPYAN than in Mn-PYAN, as evidenced by the shape measures mentioned above (Table S1, Supporting Information).^37^ Indeed, high-spin Mn(III) complexes often display Jahn–Teller distorted octahedral coordination, while high-spin Mn(II) complexes lack any LFSE.^38,39^ The *E*_1/2_ values of 652 and 567 mV vs Ag/AgCl (3 M KCl) correspond to 862 and 777 mV vs NHE for Mn-PYAN and Mn-CHXPYAN, respectively.^40^ These potentials are close to the edge (Mn-CHXPYAN) or above (Mn-PYAN) the water thermodynamic stability window marked by oxygen reduction to water (E_O2/H2O_ = +820 V vs NHE at pH 7),^41^ indicating that these complexes are likely rather resistant to oxidation in biological media.

### Ligand Protonation Constants and Stability Constants of the Mn(II) Complexes

Protonation constants of both PYAN and CHXPYAN and stability constants of their Mn(II) complexes were previously studied by Jackels et al. nearly 3 decades ago.^24,25^ However, the authors were not able to determine an exact value for the stability constant of the complex formed between Mn(II) and CHXPYAN due to their observation of other equilibria in solution. Additionally, Branco et al. later reported a slightly lower stability constant value for the Mn-PYAN complex.^42^ Therefore, we decided to repeat the determination of the protonation constants of the chelators and stability constants of metal complexes using the same ionic strength for all the systems (*I* = 0.15 M NaCl).

Chelator protonation constants (log *K*_i_^H^) defined by eq 1 were determined using pH-potentiometric titrations, which afforded four protonation constants for each chelator attributed to the protonation of the amine N atoms (Table 2, and standard deviations are shown in parentheses).
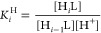
1where *i* = 1, 2...4. The first two protonation constants adopt nearly identical values for both chelators, while the third and fourth protonation constants are much lower for CHXPYAN than for PYAN, which is consistent with the results obtained by Jackels et al.^25^ The high rigidity of CHXPYAN likely forces the protonated amines to be placed at a shorter distance than in PYAN, reducing the values of log *K*_3_^H^ and log *K*_4_^H^ for the cyclohexyl derivative due to electrostatic effects. As a result, CHXPYAN displays an overall basicity lower than that of PYAN, as evidenced by the values of Σlog *K*_*i*_^H^ (*i* = 1–4). The structurally related 18-membered macrocycle 18-ane-N_6_ is characterized by a higher basicity compared with PYAN and CHXPYAN, as expected due to the increased number of amine N atoms and its higher flexibility.^43^

**Table 2:** Ligand Protonation Constants and Stability Constants and pMn Values of the Mn(II) Complexes of PYAN, CHXPYAN, 18-ane-N_6_, EDTA, CDTA, and DTPA (25°C)

	PYAN	CHXPYAN	18-ane-N_6_^43^	EDTA^4–^^48^	CDTA^4–^^48^	DTPA^5–^^44^
	0.15 M NaCl	0.15 M NaCl	0.2 M NaClO_4_	0.15 M NaNO_3_	0.15 M NaNO_3_	0.15 M NaCl
log *K*_1_^H^	9.02(1)/9.13^a^/8.99^b^	9.16(2)/9.25^c^	10.19	9.40	9.54	9.97
log *K*_2_^H^	8.27(1)/8.32^a^/8.22^b^	8.27(2)/8.57^c^	9.23	6.10	6.08	8.40
log *K*_3_^H^	6.06(1)/6.12^a^/6.03^b^	4.55(2)/4.75^c^	8.73	2.72	3.65	4.18
log *K*_4_^H^	5.18(2)/5.24^a^/5.34^b^	2.54(3)/4.03^c^	4.09	2.08	2.69	2.68
log *K*_5_^H^			2	1.23	1.14	2.01
log *K*_6_^H^			<1			1.36
Σlog *K*_i_^H^ (*i* = 1–4)	**28.52**	**24.51**	**32.24**	**20.30**	**21.97**	**25.23**
log *K*_MnL_	11.93(3)/12.5^a^/11.79^b^	12.51(3)/∼13^c^	10.5	12.46	14.32	14.54
**pMn**^d^	**7.18**	**7.41**	**5.05**	**7.83**	**9.90**	**7.95**

a Values in 0.2 M KCl from ref (24).

b Values in 0.1 M KNO_3_ from ref (42).

c Values in 0.2 M KNO_3_ from ref (25).

d pMn = −log[Mn]_free_ when [Mn^2+^] = [*L*] = 10 μM at pH = 7.4.

The stability constants of the Mn(II) complexes with PYAN and CHXPYAN defined by eq 2 were also determined through pH-potentiometric titrations.
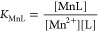
2

The log *K*_MnL_ values listed in Table 2 show that Mn-CHXPYAN has a somewhat higher stability constant than Mn-PYAN and are quite similar to that of Mn-EDTA. Both complexes have higher log *K*_MnL_ than 18-ane-N_6_, which indicates that incorporating pyridyl units into the backbone is favorable in terms of thermodynamic stability. Neither of the complexes is as stable as Mn-CDTA, though it is worth noting that the incorporation of a cyclohexyl unit into the backbone of EDTA appears to have a larger effect than in the case of the PYAN-based systems. The pMn values calculated for Mn-PYAN and Mn-CHXPYAN are lower than those obtained for the EDTA, CDTA, and DTPA^44^ complexes but considerably higher than that obtained for 18-ane-N_6_. The higher pMn value determined for the complex with CHXPYAN compared with that with PYAN is related to the lower log *K*_MnL_ value and the higher ligand basicity (Σlog *K*_i_^H^, Table 2) of PYAN.

**Figure 4:**
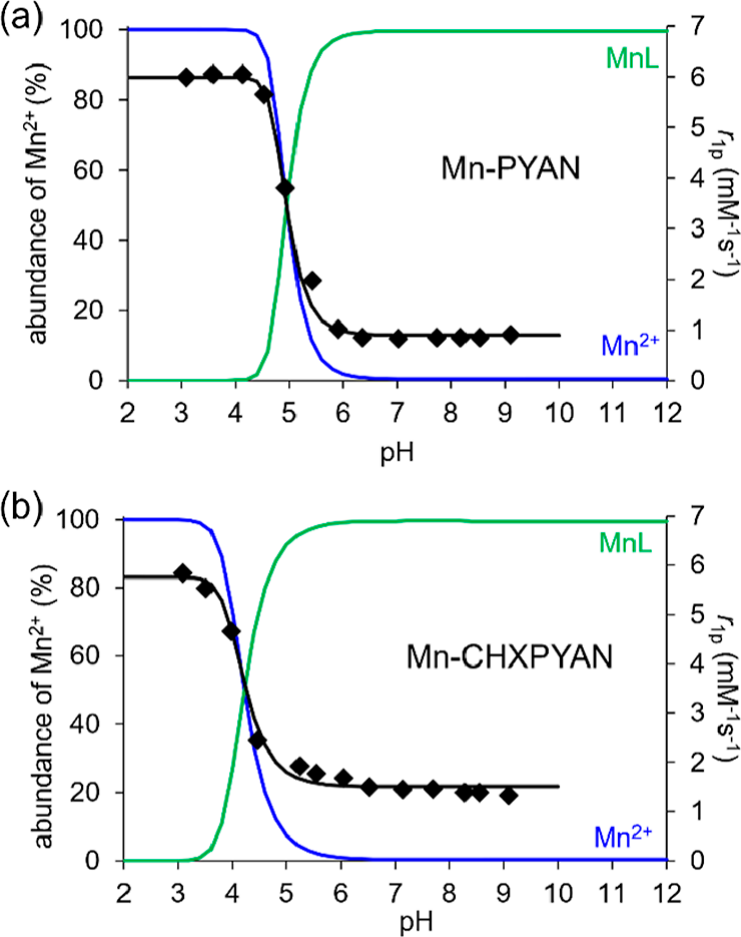
(a) Species distribution and the relaxivity values (⧫) of the Mn-PYAN system as a function of pH. ([Mn^2+^] = [PYAN] = 1.0 mM, 61 MHz, 0.15 M NaCl, 25 °C). (b) Species distribution and the relaxivity values (⧫) of the Mn-CHXPYAN system as a function of pH. ([Mn^2+^] = [CHXPYAN] = 1.0 mM, 61 MHz, 0.15 M NaCl, 25 °C).

The studies reported by Branco assumed the formation of a protonated [Mn(HL)]^3+^ species for the Mn-PYAN complex characterized by protonation constant log *K*_MnLH_ = 5.19 (*K*_MnLH_ = [MnHL]/[MnL] × [H^+^]).^42^ However, our pH-potentiometric data could be fitted very well without considering the formation of protonated species or hydroxo-complexes. In order to confirm that the equilibrium model used to fit the pH-potentiometric data was correct, we measured the relaxivity (*r*_1p_) of aqueous solutions of the complexes as a function of pH (Figure 4). Relaxivity measures the ability of a paramagnetic solute to enhance the longitudinal relaxation rate of solvent water molecules, normalized for a 1 mM concentration of the paramagnetic ion.^45^ The *r*_1p_ values measured around neutral pH values (0.83 and 1.40 mM^–1^ s^–1^ at 61 MHz and 25 °C for Mn-PYAN and Mn-CHXPYAN, respectively) are typical of complexes that lack one or more water molecules in the inner coordination sphere, the observed relaxivity being the result of the outer-sphere contribution.^46^ Relaxivity is fairly constant in the pH range of 6–9 but increases below pH ∼ 6 due to complex dissociation, reaching a relaxivity of 6.00 mM^–1^ s^–1^ at pH 3, 61 MHz, and 25 °C that is characteristic of the [Mn(H_2_O)_6_]^2+^ complex.^47^ The relaxivity profile matches the speciation diagrams calculated using the equilibrium constants obtained by pH potentiometry very well, which provides support for the equilibrium model used to fit the potentiometric data. It is worth noting that complex dissociation takes place at a lower pH for Mn-CHXPYAN than for Mn-PYAN, as expected, considering their pMn values.

### Kinetic Inertness of the Mn(II) Complexes

There is a wide consensus among the coordination chemistry community that kinetic inertness is more important for the safe use of metal complexes for imaging applications than thermodynamic stability.^8,49,50^ Thus, we conducted a detailed investigation of the transmetalation kinetics of the Mn-PYAN and Mn-CHXPYAN complexes, by following their exchange reactions with Cu^2+^ in the pH range of 2.5–4.5 (eq 3).
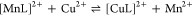
3

All reactions were carried out in the presence of a large excess of Cu^2+^ in order to guarantee the pseudo-first-order kinetic conditions. The transmetalation of Mn-PYAN in the pH range ∼2.0–4.5 is very fast, and thus the rates of the reactions were measured using the stopped-flow technique. Conversely, the exchange reactions of the Mn-CHXPYAN complex are slow and could be studied by using conventional methods. The transmetalation reactions of Mn-PYAN and Mn-CHXPYAN were monitored by following the formation of the corresponding Cu(II) complexes at 295 nm. The absorption spectra of the Mn-CHXPYAN–Cu^2+^-reacting systems as a function of time are shown in Figure S7.

**Figure 5:**
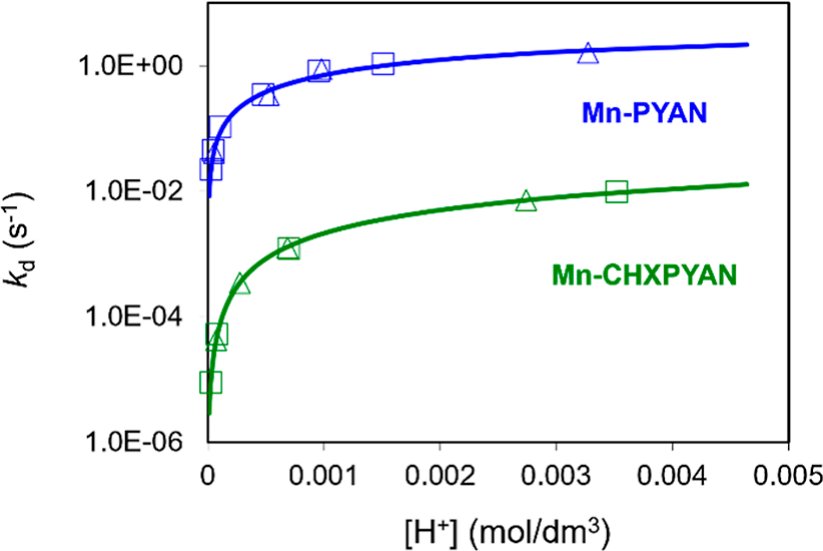
Pseudo-first-order rate constants (*k*_d_) characterizing the transmetalation reactions of Mn-PYAN and Mn-CHXPYAN with Cu^2+^ as a function of pH. [Mn-PYAN] = [Mn-CHXPYAN] = 0.1 mM, [Cu^2+^] = 1.0 mM (□), 2.0 mM (△), 0.15 M NaCl, 25 °C.

As shown in Figure 5, the *k*_d_ values exhibit a similar dependence in the reactions of Mn-PYAN and Mn-CHXPYAN with Cu^2+^. The *k*_d_ values increase upon increase in [H^+^] and are independent of the concentration of Cu^2+^ as it was found for Mn-PCTA, Mn-PC3AM, and Mn-bispidine complexes.^49,51^ Considering these results, it can be assumed that the rate-determining step of the transmetalation reactions is the dissociation of the Mn(II) complexes followed by the fast reaction between the free ligands and the Cu^2+^ ion. The increase in the *k*_d_ values with increasing H^+^ concentration can be interpreted in terms of the extremely slow spontaneous (*k*_0_, eq 4) and the proton-assisted (k_*[Mn(HL)_, eq 6) dissociation of Mn(II) complexes. The saturation behavior of the plots of *k*_d_ versus [H^+^] (Figure 5) is characteristic for the equilibrium formation and the accumulation of the *[Mn(HL)] intermediate characterized by the *K_H_ protonation constant (eq 5). The analysis of the rate constants obtained for Mn-CHXPYAN indicates a second-order dependence of *k*_d_ on [H^+^], which can be explained by the proton-assisted dissociation of the *[Mn(HL)] intermediate (*k*^H^_*[Mn(HL)]_, eq 7).
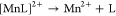
4
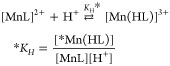
5
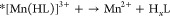
6
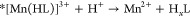
7

In the presence of excess of the exchanging ion, the transmetalation can be treated as a pseudo-first-order process, and the rate of reactions can be expressed with eq 8, where *k*_d_ is a pseudo-first-order rate constant and [MnL]_*t*_ is the total concentration of the complex.
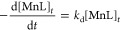
8

Through consideration of all the possible pathways, the rate of the transmetalation of Mn-PYAN and Mn-CHXPYAN with Cu^2+^ can be expressed by eq 9
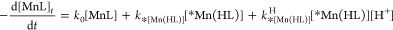
9Considering the total concentration of the Mn(II) complex ([MnL]_*t*_ = [MnL] + [*Mn(HL)], as well as the equation defining the protonation constant for the formation of the *[Mn(HL)] intermediate (eq 5) and eq 8, the pseudo-first-order rate constant can be expressed as follows
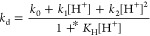
10where *k*_1_ = k_*[Mn(HL)]_ × *K_H_ and *k*_2_ = *k*^H^_*[Mn(HL)]_ × *K_H_. The rate and protonation constants characterizing the transmetalation of Mn-PYAN and Mn-CHXPYAN with Cu^2+^ have been calculated by fitting the *k*_d_ values presented in Figure 5 to eq 10 and the values obtained are compared with the corresponding values of Mn-DOTA, Mn-DO3A, Mn-PCTA, Mn-PC3AM^H^, and Mn-bispidine in Table 3.

**Table 3:** Rate and Equilibrium Constants and Half-Life Values Characterizing the Dissociation Reactions of Mn-PYAN, Mn-CHXPYAN, Mn-DOTA, Mn-PCTA, Mn-PC3AM, and Mn-Bispidine (25 °C)

	PYAN	CHXPYAN	DOTA^4–^^52^	DO3A^3–^^51^	PCTA^3–^^51^	PC3AM^H^^51^	bispidine^3–^^49^
***I***	0.15 M NaCl		0.1 M Me_4_NCl	0.15 M NaCl	0.15 M NaCl	0.15 M NaCl	0.1 M KCl (37 °C)
*k*_0_ (s^–1^)			1.8 × 10^–7^		7.0 × 10^–2^		
*k*_1_ (M^–1^ s^–1^)	827 ± 54	0.23 ± 0.03	4.0 × 10^–2^	0.45	0.11	1.7 × 10^–2^	1.6 × 10^–3^
*k*_2_ (M^–2^ s^–1^)		(6.4 ± 0.8) × 10^3^	1.6 × 10^3^	3.2 × 10^2^	3.5 × 10^2^		5.0 × 10^–4^
*k*_3_^Zn^ (M^–1^ s^–1^)			1.5 × 10^–5^				
log*K_H_	2.23 (4)	3.33 (5)	4.26				
*k*_d_ (s^–1^) at pH = 7.4	3.3 × 10^–5^	9.0 × 10^–9^	1.8 × 10^–7^^a^	1.8 × 10^–8^	3.3 × 10^–9^	4.3 × 10^–10^	6.4 × 10^–11^
*t*_1/2_ (h) at pH = 7.4	**5.85**	**2.13** × **10**^**4**^	**1.04** × **10**^**3**^^a^	**1.1** × **10**^**4**^	**5.9** × **10**^**4**^	**4.5** × **10**^**5**^	**3.0** × **10**^**6**^

a Calculated using a Zn^2+^ concentration of 10^–5^ M.

The fits of the data resulted in negligible values of *k*_0_, indicating that spontaneous dissociation does not contribute to the dissociation of the Mn(II) complex under the conditions investigated here. The value of *k*_1_ determined for Mn-CHXPYAN is 3600 times lower than that determined for Mn-PYAN, highlighting the beneficial impact that incorporating rigid cyclohexyl units has on kinetic inertness. The value of *k*_1_ obtained for Mn-CHXPYAN is between the values reported for Mn-DOTA and Mn-DO3A.^51,52^ The experimental rate constants were used to estimate the half-lives (*t*_1/2_) of the Mn(II) complexes close to physiological conditions (pH 7.4 and 25 °C). Mn-PYAN behaves poorly in terms of kinetic inertness; it is the most labile system of those reported here with the shortest half-life at pH 7.4 of 5.85 h. However, Mn-CHXPYAN presents high kinetic inertness, with a value of *t*_1/2_ at pH 7.4 being one magnitude higher than that of Mn-DOTA, almost double than that of Mn-DO3A and half than that of Mn-PCTA. While Mn-PC3AM^H^ and Mn-bispidine are more inert,^49,51^ the behavior of Mn-CHXPYAN is still quite promising, considering that Mn(II) complexes are in many cases quite kinetically labile. Furthermore, the inertness of Mn-CHXPYAN compares well with that of the Mn-DOTA and Mn-DO3A complexes, a very promising characteristic, as the manganese-52 complexes of DOTA and DO3A were reported to offer good stability in vivo.^53^ Of note, the Mn-DTPA complex is extremely labile, as complex dissociation in the presence of Cu^2+^ is already complete at the dead time of the stopped-flow technique (∼2 ms).^44^

### Radiolabeling

Radiolabeling conditions were optimized for both PYAN and CHXPYAN with [^52^Mn]Mn(II). Efficient radiolabeling of CHXPYAN was observed at pH 7 when the complex was incubated at 37 °C for 1 h with radiochemical yields (RCYs) of >95% obtained at 80 MBq/μmol. However, radiolabeling of PYAN required 2 h incubation at pH adjustment to 8.5 to obtain >95% RCY (60 MBq/μmol). The results were confirmed with iTLC with the chromatograms shown in the Supporting Information (Figure S8). The molar activities reported herein, for example, for CHXPYAN (0.08MBq/nmol), are comparable to the ones reported in our previous work for chelators (Oxo-DO3A, DO3A, and DOTA—0.17 MBq/nmol).^53^

### In Vitro Stability Studies

Assessment of the stability and kinetic inertness of the radiocomplexes before in vivo evaluation was conducted to assess the possibilities of transchelation. In vitro stability assays including incubating the radiocomplexes with an excess (100 μM) of biologically relevant metal ions (Mg^2+^, Zn^2+^, Fe^2+^, and Cu^2+^), DTPA, and mouse and human serum were conducted.

**Figure 6:**
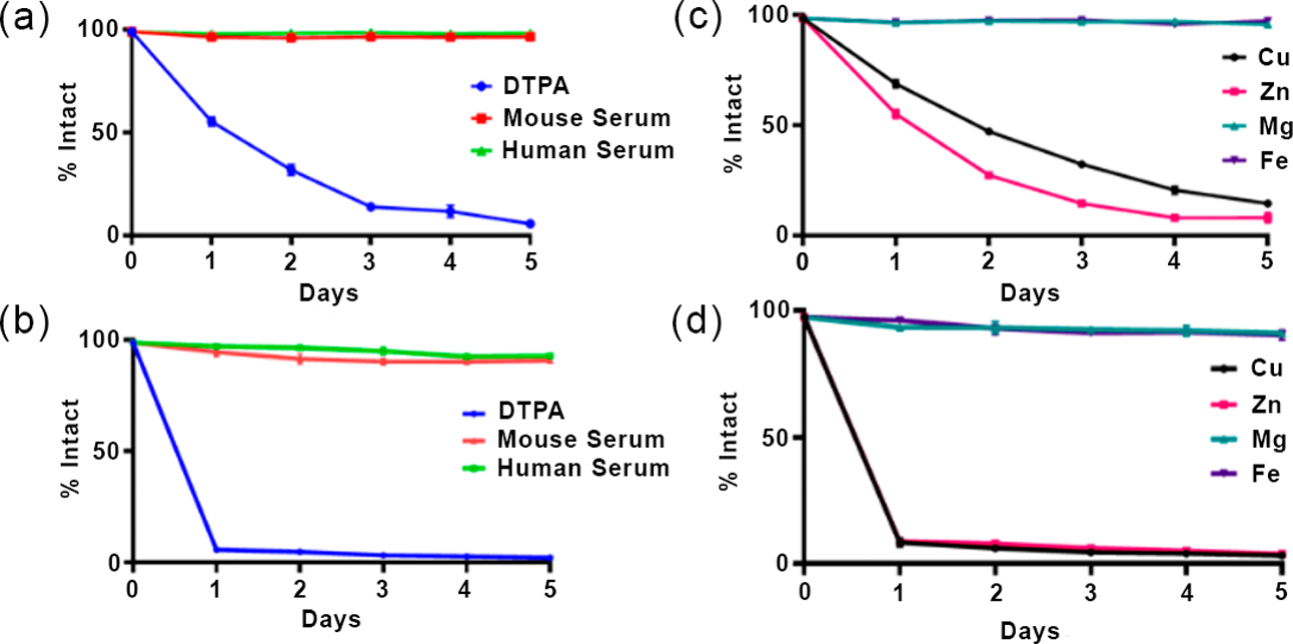
Stability studies of (a) [^52^Mn]Mn-CHXPYAN and (b) [^52^Mn]Mn-PYAN in DTPA (5 equiv), human and mouse. Stability studies of (c) [^52^Mn]Mn-CHXPYAN and (d) [^52^Mn]Mn-PYAN in 10 molar excess of 100 μM of Cu^2+^, Zn^2+^, Fe^2+^, and Mg^2+^ over 5 days (Each data point is *n* = 3; error bars are smaller than symbol size, see Figure S13). The final pH of metal and DTPA challenge samples was ∼5.5, where [*Mn(HL)] is the concentration of the protonated intermediate.

Human and mouse serum stability of [^52^Mn]Mn-CHXPYAN showed a 98.04 ± 0.18 and 96.31 ± 1.34% intact complex at 5 days postincubation, respectively. The radioactivity associated with the protein pellet was <15%, with no significant differences between the time points (Tables S5 and S6, Supporting Information), indicating that [^52^Mn]Mn-CHXPYAN is stable in both mouse and human serum. In the presence of DTPA, gradual decomplexation was observed with the complex being 5.73 ± 0.25% intact on day 5 after incubation (Figure 6a). [^52^Mn]Mn-PYAN remained stable in human serum with a 92.78 ± 1.22% intact complex, but the stability decreased slightly to a 90.67 ± 1.12% intact complex in mouse serum after 5 days of incubation. Decomplexation of [^52^Mn]Mn-PYAN in the presence of DTPA was significant with 2.32 ± 0.66% complex intact on day 1 after incubation (Figure 6b).

Figure 6c,d shows a summary of the results of the stability studies of the radiocomplexes in the presence of a 10 molar excess of biologically relevant metal ions (Mg^2+^, Zn^2+^, Fe^2+^, and Cu^2+^). Both complexes remained stable in the presence of Fe^2+^ and Mg^2+^ with >95% for over 5 days but decomplex in the presence of Zn^2+^ and Cu^2+^. Decomplexation of [^52^Mn]Mn-CHXPYAN was gradual in the presence of Cu (14.56 ± 1.09% intact complex) and Zn (8.14 ± 1.76% intact complex) on day 5 (Figure 6c), while decomplexation of [^52^Mn]Mn-PYAN in the presence of Zn and Cu was rapid with a <4% intact complex on day 1 upon incubation (Figure 6d). The radio-TLC chromatograms for all in vitro assays are shown in the Supporting Information (Figures S9–S12). It must be stressed that the addition of the competitor metal ions and DTPA decreases the pH of the mixture to about ∼5. This very likely triggers the dissociation of the complex following the acid-catalyzed dissociation pathway, as our kinetic studies evidence that Cu^2+^ has no significant effect in the rates of dissociation (see above). By taking into account the acid-catalyzed dissociation rate at pH = 5.5 (*k*_d_ = 7.9 × 10^–7^ s^–1^, *t*_1/2_ = 10 days at 25 °C) and about 3–4 times faster decomplexation reaction at 37 °C, the dissociation rate of [^nat/52^Mn]Mn-CHXPYAN determined by the transmetalation reactions with Cu^2+^ and obtained in the in vitro stability studies is in good agreement. On the other hand, the results obtained for the radiolabeled complexes evidence that [^52^Mn]Mn-CHXPYAN is considerably more inert than [^52^Mn]Mn-PYAN.

### In Vivo Studies

In vitro stability studies involving significantly higher concentrations of metal ions occur under extreme conditions and do not always reflect the actual biological conditions in vivo.^54,55^ To assess the in vivo stability of these radiocomplexes, we conducted an in vivo biodistribution assay to determine their clearance routes and uptake profiles with a comparison to that of nonbound [^52^Mn]Mn(II).

**Figure 7:**
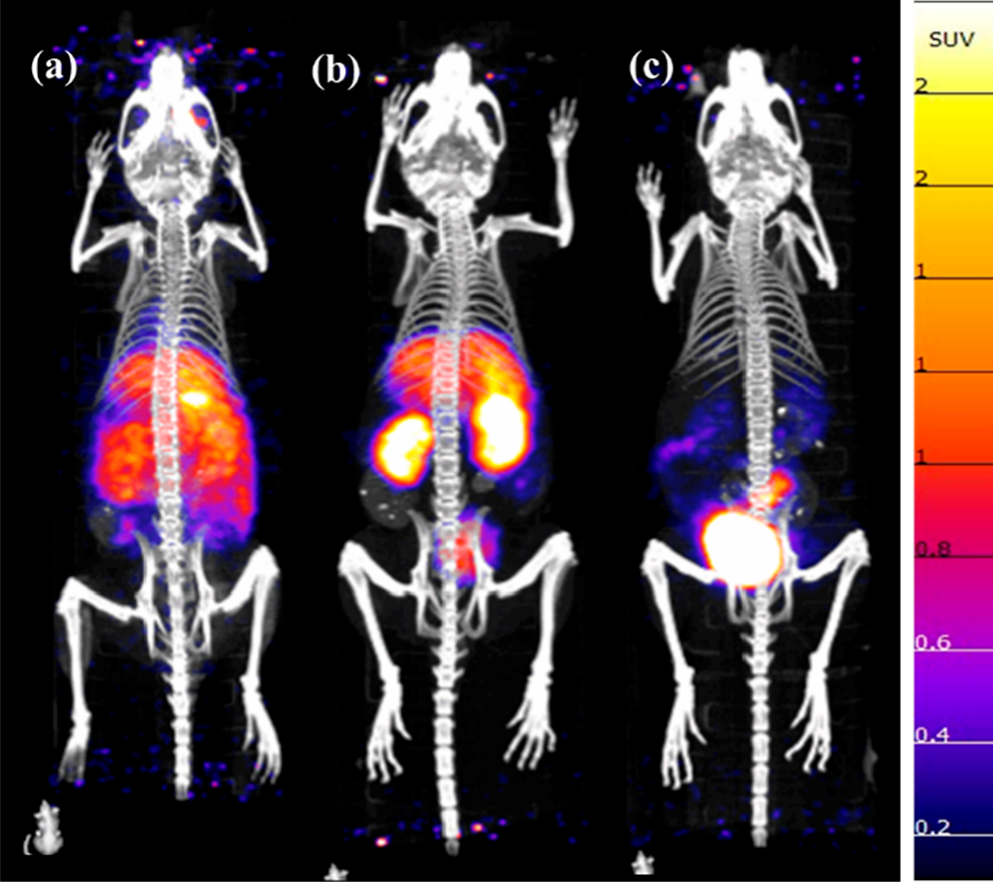
PET images of healthy mice 1.5 h postinjection of (a) [^52^Mn]MnCl_2_, (b) [^52^Mn]Mn-PYAN, and (c) [^52^Mn]Mn-CHXPYAN (*n* = *4*). The images are shown on the same scale.

[^52^Mn]Mn-CHXPYAN and [^52^Mn]Mn-PYAN radiocomplexes were evaluated in vivo through 90 min dynamic PET/CT imaging of heathy mice followed by an ex vivo biodistribution analysis. Similarly, [^52^Mn]MnCl_2_ was also studied as a control. Figure 7 shows the maximum intensity projection images for [^52^Mn]MnCl_2_ (Figure 7a), [^52^Mn]Mn-PYAN (Figure 7b), and [^52^Mn]Mn-CHXPYAN (Figure 7c), shown on the same scale.

**Figure 8:**
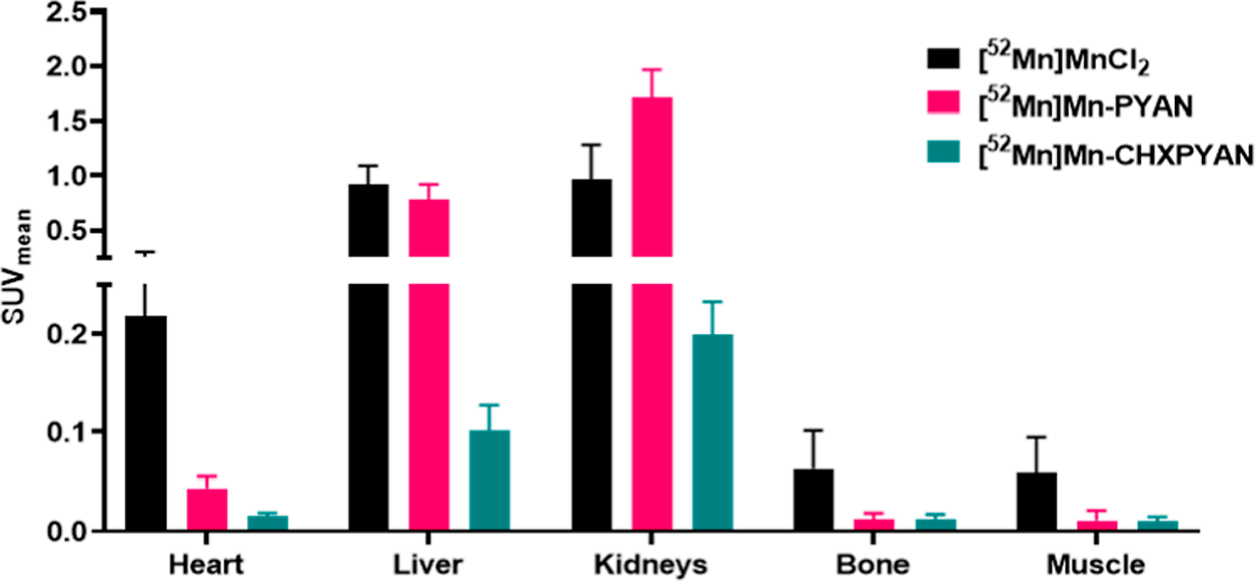
SUV_mean_ analysis in selected tissues of interest 1.5 h postinjection.

Regions of interest (ROIs) were manually drawn using CT images to determine the standard uptake value (SUV_mean_) using VivoQuant imaging software. SUV mean analysis showed a significantly lower concentration of [^52^Mn]Mn-CHXPYAN in the liver (SUV_mean_ 0.10 ± 0.02) and kidneys (SUV_mean_ 0.20 ± 0.03) compared to the concentration of [^52^Mn]Mn-PYAN in the liver (SUV_mean_ 0.77 ± 0.14) and kidneys (SUV_mean_ 1.70 ± 0.26) at 1.5 h postinjection, correlating well with the PET images. Figure 8 shows SUV mean analysis for selected tissues of interest including heart, kidneys, and liver where unchelated ^52^Mn is known to localize.

**Figure 9:**
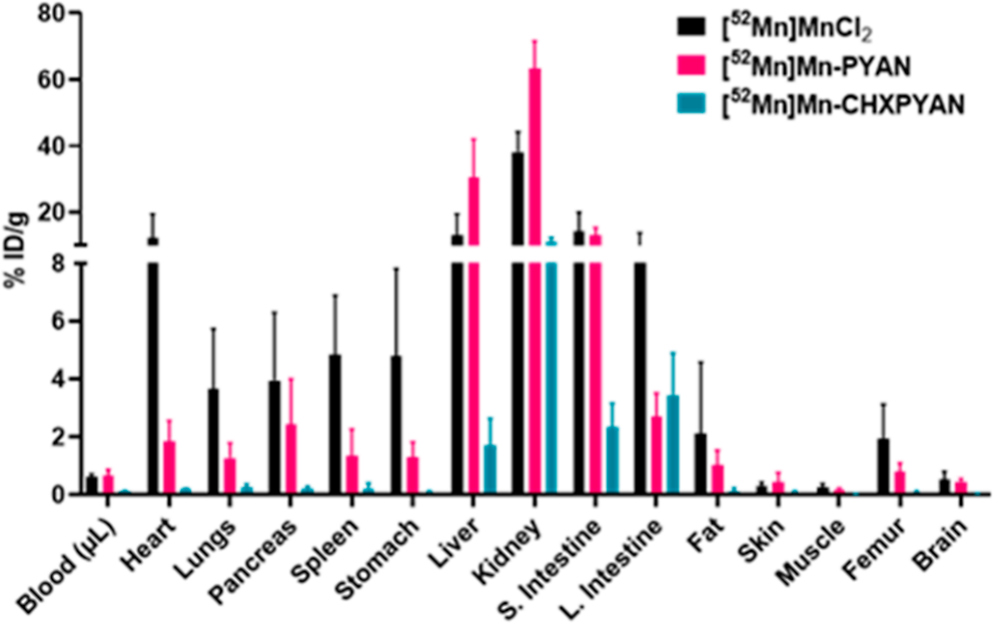
Biodistribution of [^52^Mn]MnCl_2_, [^52^Mn]Mn-PYAN, and [^52^Mn]Mn-CHXPYAN in healthy mice 1.5 h postinjection (*n* = *4*).

PET images in Figure 7 are consistent with the biodistribution data (Figure 9). Both radiocomplexes showed excretion through the renal pathway with [^52^Mn]Mn-CHXPYAN clearing faster (kidneys: 11.27 ± 1.11% ID/g) compared to [^52^Mn]Mn-PYAN (kidneys: 63.34% ID/g) that showed persistent accumulation of radioactivity.

Detailed biodistribution data are provided in the Supporting Information (Table S4). Stable radiocomplexes are rapidly eliminated through the hepatobiliary or digestive system, whereas unstable radiocomplexes tend to have a biodistribution pattern similar to unchelated radiometals, resulting in slow elimination from the respective organs.^56,57^ In vivo biodistribution studies of [^52^Mn]MnCl_2_ in mice have shown uptake and accumulation of radioactivity in organs such as the heart (16.01 ± 0.1% ID/g), liver (9.6 ± 1.9% ID/g), kidney (29.9 ± 9.6% ID/g), spleen (4.2 ± 0.6% ID/g), and pancreas (4.1 ± 1.1% ID/g).^53,58^

[^52^Mn]Mn-CHXPYAN showed a different biodistribution profile from that of [^52^Mn]MnCl_2_ with fast clearance from the liver and kidneys 1.5 h postinjection (liver: 1.72 ± 0.93% ID/g; kidneys: 11.27 ± 1.11% ID/g). These results are similar or show slightly better clearance than those reported by Omweri et al. for [^52^Mn]Mn-Oxo-DO3A (liver: 2.3 ± 0.2% ID/g; kidneys: 25.4 ± 4.8% ID/g) and [^52^Mn]Mn-DO3A (liver: 5.3 ± 0.5% ID/g; kidneys: 14.5 ± 3.5% ID/g) 1 h postinjection.^53^ [^52^Mn]Mn-PYAN showed a similar distribution pattern to that of unchelated [^52^Mn]MnCl_2_ with persistent accumulation of radioactivity in the liver, pancreas, and spleen. These are organs in which free [^52^Mn]Mn(II) is known to accumulate, and this may suggest some degree of decomplexation.

## Conclusions

We have demonstrated that CHXPYAN is a very promising platform for the development of ^52^Mn-based radiopharmaceuticals. Comparison of the Mn(II) complex of this chelator with that of its more flexible analogue, PYAN, shows that the incorporation of two cyclohexyl units into the 18-membered backbone confers increased thermodynamic stability and kinetic inertness. Interestingly, the increased rigidity of Mn-CHXPYAN facilitates the oxidation of Mn(II) to Mn(III), likely due to the more symmetrical distribution of the donor atoms in the coordination sphere, evidenced in the crystal structures of the complexes.

These results translated well to the radiochemical studies with CHXPYAN showing superior radiolabeling and slightly better stability in the in vitro assays. Most importantly, in vivo biodistribution and PET/CT images showed that CHXPYAN was far superior to PYAN, remaining stable in vivo. In conclusion, this work is an important contribution to the development of new bifunctional systems for Mn(II)-based radiopharmaceuticals, which are currently under development by introducing coupling functions through a secondary amine N atom or a pyridyl ring.

## Experimental Section

### General Considerations

Mass spectra were recorded on an LTQ-Orbitrap Discovery mass spectrometer coupled to a Thermo Accela HPLC instrument in ESI positive mode. Flash chromatography purifications were carried out using a puriFlash XS 420 InterChim chromatographer equipped with a UV-DAD detector and a 20 g BGB Aquarius C18AQ reverse phase column (100 Å, spherical, 15 μm), using H_2_O and CH_3_CN as mobile phases (flow rate 15 mL/min). Analytical HPLC analysis of the stable Mn(II) complexes was performed using a Jasco LC-4000 instrument equipped with a UV-4075 detector, equipped with a Hypersil GOLD aQ column (5 μm, 100 × 4.6 mm) and H_2_O and CH_3_CN + 0.1% formic acid as the mobile phases, operating at a flow rate of 0.3 mL/min. Semipreparative HPLC was carried out on the same apparatus, equipped with a Fortis C18 column (5 μm, 250 × 10 mm). Aqueous solutions of the final compounds were lyophilized in a Biobase BK-FD10 Series Vacuum Freeze-Dryer.

### Synthesis of the Mn(II) Complexes

The solvents and reagents were of reagent grade and were purchased from commercial sources and used without further purification. Chelators PYAN and CHXPYAN were prepared according to previously reported procedures.^24,26^ Both chelators were purified by semipreparative HPLC using H_2_O and CH_3_CN + 0.1% formic acid as the mobile phases to ensure sufficient purity for radiolabeling experiments (Table S2, Supporting Information).

### [Mn(PYAN)](NO_3_)_2_

PYAN (50.0 mg, 0.153 mmol) is suspended in EtOH (5 mL) and Mn(NO_3_)_2_ (48.7 mg, 194 mmol) is added. Once the metal salt is added, a clear yellow solution forms, which is refluxed for 30 min. The solvent is evaporated, and the resulting residue is purified by flash chromatography (compound elutes in 100% H_2_O with a retention time of 3.5 min or 2.56 CV). The fractions of interest were lyophilized to give an off-white solid (53.0 mg, yield = 69%). Experimental HR-MS (ESI^+^,% BPI): *m*/*z* 190.5793 (100), 443.1472 (63), 380.1516 (36); calculated for [C_18_H_26_MnN_6_]^2 +^ 190.5794, calculated for [C_18_H_25_MnN_6_]^+^ 380.1516, and calculated for [C_18_H_26_MnN_7_O_3_]^+^ 443.1472.

### [Mn(CHXPYAN)](NO_3_)_2_

To a suspension of CHXPYAN (50.2 mg, 0.116 mmol) in EtOH (5 mL) was added Mn(NO_3_)_2_ (36.6 mg, 149 mmol), resulting in a bright yellow solution which is refluxed for 15 min. The solvent is evaporated, and the resulting residue is purified by flash chromatography (compound elutes in 100% H_2_O with a retention time of 12.9 min or 9.40 CV). The fractions of interest were lyophilized to give an off-white solid (34,6 mg, yield = 49%). Experimental HR-MS (ESI^+^,% BPI): *m*/*z* 224.6261 (100), 551.2409 (61); calculated for [C_26_H_38_MnN_6_]^2 +^ 224.6264, calculated for [C_26_H_34_MnN_7_O_3_]^+^ 551.2411.

### Electrochemical Measurements

Cyclic voltammetry measurements were performed with a three-electrode configuration on an Autolab PGSTAT302 M potentiostat-galvanostat. A glassy-carbon disk (Metrohm 61204600) was used as the working electrode. The surface of this electrode was polished with α-Al_2_O_3_ (0.3 μm) and washed with distilled water before every measurement. The reference electrode used was a Ag/AgCl reference electrode filled with 3 M KCl (Metrohm 6.0726.100). A Pt wire was used as the counter electrode. The complex solutions (2 mM) containing 0.15 M were deoxygenated by bubbling nitrogen before each measurement.

#### Solution Thermodynamic Studies

##### Materials

The chemicals used for the experiments were of the highest analytical grade. The concentrations of the MnCl_2_ solutions were determined by complexometric titration with standardized Na_2_H_2_EDTA and Eriochrome Black T as indicators. The concentration of the PYAN and CHXPYAN was determined by pH-potentiometric titration in the presence and absence of a large (40-fold) excess of CaCl_2_. The pH-potentiometric titrations were performed with standardized 0.2 M NaOH.

##### pH-Potentiometric Measurements

The protonation constants of PYAN and CHXPYAN and the stability constants of Mn(II) complexes were determined by pH-potentiometric titrations. The metal-to-ligand concentration ratio was 1:1 (the concentration of the ligand was generally 0.002 M). For the pH measurements and titrations, a Metrohm 888 Titrando titration workstation and Metrohm-6.0234.110 combined electrode was used. Equilibrium measurements were carried out at a constant ionic strength (0.15 M NaCl) in 6 mL samples at 25 °C. The solutions were stirred, and N_2_ was bubbled through them. The titrations were made in the pH range of 1.7–12.0. KH-phthalate (pH = 4.005) and borax (pH = 9.177) buffers were used to calibrate the pH meter. For the calculation of [H^+^] from the measured pH values, the method proposed by Irving et al. was used as follows.^59^ A 0.01 M HCl solution was titrated with a standardized NaOH solution at 0.15 M NaCl ionic strength. The differences (A) between the measured (pH_read_) and calculated pH (−log[H^+^]) values were used to obtain the equilibrium H^+^ concentration from the pH values measured in the titration experiments (*A* = 0.04). For the equilibrium calculations, the stoichiometric water ionic product (p*K*_w_) was also needed to calculate the [OH^–^] values under basic conditions. The *V*_NaOH_ – pH_read_ data pairs of the HCl–NaOH titration obtained in the pH range of 10.5–12.0 were used to calculate the p*K*_w_ value (p*K*_w_ = 13.77). The protonation and stability constants were calculated with the PSEQUAD program.^60^

##### ^1^H NMR Relaxometry

The relaxivity values were calculated from the longitudinal relaxation time of H_2_O protons (*T*_1_) measured at 61 MHz on a Stelar relaxometer connected to a Bruker WP80 NMR electromagnet adapted to variable-field measurements (15–80 MHz proton Larmor frequency). The temperature of the sample holder was controlled with a thermostated air stream. The longitudinal relaxation time was measured with the “inversion recovery” method (180°–τ–90°) by using 12 different τ values with a typical 90° pulse width of 10.7 μs, 4 scans. The measurements were performed with 1 mM solution of the Mn^2+^ and PYAN or CHXPYAN, so the relaxivity values were given as *r*_1_ = 1/*T*_1p_ + 1/*T*_1w_ where *T*_1p_ and *T*_1w_ were the relaxation times of bulk water protons in the presence and absence of paramagnetic species. The variable-pH relaxivity measurements of Mn^2+^-PYAN and Mn^2+^-CHXPYAN systems could be carried out by direct titration of the samples in the pH range of 3.0–9.0 (61 MHz and 25 °C). The pH was adjusted by stepwise addition of a concentrated NaOH or HCl solution.

### Transmetalation Studies

Rates of the metal exchange reactions of Mn-PYAN and Mn-CHXPYAN with Cu^2+^ were measured by spectrophotometry by following the formation of the related Cu(II) complexes at 295 nm with an Applied Photophysics DX-17MV stopped-flow instrument and PerkinElmer Lambda 365 UV–vis spectrophotometer using 1.0 cm cells, respectively. The concentration of Mn-PYAN and Mn-CHXPYAN was 1.0 × 10^–4^ M, whereas the concentration of Cu^2+^ was 10 and 20 times higher in order to guarantee pseudo-first-order conditions. The temperature was maintained at 25 °C and the ionic strength of the solutions was kept constant with 0.15 M NaCl. The exchange rates were studied in the pH range of *ca*. 2.0–4.5. To keep the pH values constant, monochloroacetic acid (pH range 2.0–3.3), 1,4-dimethylpiperazine (pH range 3.3–4.1), and *N*-methylpiperazine (pH range 4.1–4.5) buffers were used (0.01 M). The pseudo-first-order rate constants (*k*_d_) were calculated by fitting the absorbance data to eq 11.
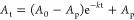
11where *A*_t_, *A*_0_, and *A*_p_ are the absorbance values at time t, at the start of the reaction, and at equilibrium, respectively. The calculation of the kinetic parameters was performed by the fitting of the absorbance–time data pairs with the Micromath Scientist computer program (version 2.0, Salt Lake City, UT, USA).

### Crystal Structure Determinations

Addition of KPF_6_ to an aqueous solution of each complex resulted in immediate precipitation of the complex, which was redissolved by adding acetonitrile to the suspension. Slow evaporation of the acetonitrile contained in the mixture afforded either colorless prisms, in the case of Mn-CHXPYAN, or a mixture of colorless prisms and needles, in the case of Mn-PYAN, adequate for X-ray diffraction. The prisms of Mn-CHXPYAN present an asymmetric unit consisting of two units of the Mn(II) complex and four PF_6_^–^ anions. The prisms of Mn-PYAN present an asymmetric unit consisting of one unit of the Mn(II) complex, half a PF_6_^–^ anion, one and a half NO_3_^–^ anions, and two water molecules. The needles of Mn-PYAN present an asymmetric unit consisting of three units of the Mn(II) complex, six PF_6_^–^ anions, and one water molecule.

Single crystals of [Mn(PYAN)](PF_6_)_0,5_(NO_3_)_1,5_, [Mn(PYAN)](PF_6_)_2_, and [Mn(CHXPYAN)](PF_6_)_2_ were analyzed by X-ray diffraction. Crystallographic data and the structure refinement parameters are given in Table S3, Supporting Information. Measurements were performed on a Bruker D8 VENTURE diffractometer with a Photon 100 CMOS detector at 100 K and Mo–Kα radiation (λ = 0.71073 Å) generated by an Incoatec high brillance microfocus source equipped with Incoatec Helios multilayer optics. The software APEX^61^ was used for collecting frames of data, indexing reflections, and the determination of lattice parameters, SAINT^62^ for integration of intensity of reflections, and SADABS^63^ for scaling and empirical absorption correction. The structure was solved by dual-space methods using the program SHELXT.^64^ All non-hydrogen atoms were refined with anisotropic thermal parameters by full-matrix least-squares calculations on *F*^2^ using the program SHELXL-2014.^65^ Hydrogen atoms of the compound were inserted at calculated positions and constrained with the isotropic thermal parameters. CCDC 2335672–2335674 contains the supplementary crystallographic data, which can be obtained free of charge from the Cambridge Crystallographic Data Centre via www.ccdc.ac.uk/data_request/cif.

#### Radiochemistry

##### Materials and Experiments

Natural chromium powder (5 N purity) was purchased from ESPI metals (Ashland, OR). 1 mL solid-phase extraction (SPE) tubes (1 mL) with frits were sourced from Millipore Sigma (Burlington, MA). AG1-X8 analytical-grade 200–400 mesh chloride-form resin was obtained from Bio-Rad (Hercules, CA). Proton bombardments were performed on a TR-24 cyclotron [Advanced Cyclotron Systems Inc. (ACSI Inc.)]. Activity measurements were performed on a Capintec dose calibrator (CRC-25R) Capintec Inc. (Florham Park, NJ, USA) cross-calibrated with a Canberra GC2018 High Purity Germanium detector (HPGe) with a DSA = 100 multichannel analyzer (Meriden, CT, USA). Radio-TLC was performed on iTLC SG paper purchased from Agilent Technologies (Santa Clara, CA, USA) and scanned using an AR-2000 Imaging Scanner (Eckert and Ziegler, MA, USA) and processed using WinScan Radio-TLC software (Eckert and Ziegler, AR-2000, WinScan software, Berlin, Germany).

##### Production and Purification of ^52^Mn

Production and purification of ^52^Mn followed a published protocol by Omweri et al.^53^ Briefly, pressed natural chromium powder pellets were irradiated with 12.5 MeV protons at 15 μA for 4 h. ^52^Mn was separated from the target material by employing three-sequential anion exchange purifications.^18^

AG1-X8 resin packed in 1 mL SPE tubes was prepared according to Pyles et al.^15^ and [^52^Mn]MnCl_2_ eluted in 6 M HCl.

##### Radiolabeling

Chelators CHXPYAN and PYAN were dissolved in Milli-Q water to make 2.5 nM stock solutions. Radiolabeling conditions for ^52^Mn were optimized by analyzing varying concentrations of CHXPYAN and PYAN chelators (2.3–46 nmol). All radiolabeling studies were conducted with buffered (1x PBS) [^52^Mn]MnCl_2_, pH 7. For CHXPYAN, varying chelator concentrations were combined with 1.85 MBq (50 μCi) of buffered [^52^Mn]MnCl_2_ and pH adjusted to 7 in 100 μL. The reaction mixtures were incubated at 37 °C and 800 rpm for 1 h. Similarly, varying concentrations of PYAN were combined with the same amount of activity and pH adjusted to 8.5 and the reaction mixtures were incubated for 2 h at 37 °C. Radiolabeling yields were assessed by iTLC-SG in sodium citrate (0.1 M, pH 5) as the mobile phase.

##### Serum Stability

The ^52^Mn radiocomplexes were incubated with human or mouse serum at 37 °C. 50 μL of the radiocomplexes in PBS was combined with 500 μL of serum in triplicate and incubated at 37 °C for over 5 days. At indicated time points, 50 μL of the reaction mixture was removed and proteins were precipitated by addition of 50 μL of methanol. The precipitated proteins were centrifuged before iTLC measurements were performed over a period of 5 days on the supernatant. All the radio-TLC chromatograms for evaluation of stabilities are available in the Supporting Information section as Figures S9–S12.

##### Metal and DTPA Metal Challenges

For metal challenge studies, a 10 molar excess of 100 μM of metal ions in chloride solutions including CuCl_2_, ZnCl_2_, FeCl_2_, and MgCl_2_ relative to ligand concentration was incubated with the radiocomplexes at 37 °C. A 5 molar excess of DTPA was also added to compete with ^52^Mn in the radiocomplexes. Decomplexation was monitored by radio-iTLC over a period of 5 days.

##### In Vivo Biodistribution and Imaging Studies

All animal studies conducted in this work were performed using a protocol approved by the Institutional Animal Care and Use Committee (IACUC) at the University of Alabama at Birmingham and were compliant with national animal welfare policies and guidelines. Female BALB/c mice were obtained from Charles River Laboratories (Charles River, Wilmington, MA). The animals were acclimatized for 1 week before initiation of studies.

[^52^Mn]Mn-CHXPYAN and [^52^Mn]Mn-PYAN were synthesized with molar specific activities of 80 and 60 MBq/μmol, respectively. 1.04 ± 0.07 MBq (28 ± 2 μCi) of the radiocomplexes in 100 μL injection doses was prepared. The mice (n = 4 per group) were anesthetized with 2.5% isoflurane in oxygen and were injected via the retroorbital sinus. For comparison purposes, 1.04 ± 0.07 MBq (28 ± 2 μCi) of [^52^Mn]MnCl_2_, pH 7 was also injected. After injection, mice were imaged on a Sofie GNEXT PET/CT scanner for a 90 min dynamic scan (9 frames of 10 min each) followed by a 3 min CT at 80 kVp for anatomical reference. PET images were reconstructed via 3D-OSEM (Ordered Subset Expectation Maximization) algorithm (24 subsets and 3 iterations), with random, attenuation, and decay correction, and CT was reconstructed with the modified Feldkamp algorithm and analyzed using VivoQuant 4.0 (Invicro Imaging Service and Software, Boston MA) software. After imaging, mice were euthanized, and organs were collected, weighed, and counted for associated radioactivity on an automated gamma counter. Radioactivity uptake was calculated as the percent injected dose per gram of tissue (% ID/g). Following the reconstruction of the images, ROIs (heart, liver, kidneys, muscle, and bone) were hand-drawn to determine the SUVs using the VivoQuant imaging software.

## Supporting Information Available

MS, HPLC traces, plots of the linear dependence of anodic and cathodic peak currents, spectrophotometric kinetic experiments, HPLC purification methods, crystal data, and refinement details (PDF)
Crystallographic data of [Mn(PYAN)](PF_6_)_0,5_(NO_3_)_1,5_ (CIF)
Crystallographic data of [Mn(PYAN)](PF_6_)_2_ (CIF)
Crystallographic data of [Mn(CHXPYAN)](PF_6_)_2_ (CIF)
Molecular formula strings (CSV)

## Supplementary Material

jm4c00812_si_001.pdf

jm4c00812_si_002.cif

jm4c00812_si_003.cif

jm4c00812_si_004.cif

## Abbreviations

BPI: base peak intensity
CCDC: Cambridge Crystallographic Data Centre
CDTA: 2,2′,2″,2‴-{[(1*R*,2*R*)-cyclohexane-1,2-diyl]bis(azanetriyl)}tetraacetate
CT: computed tomography
DO3A: 1,4,7,10-tetraazacyclododecane-1,4,7-triacetate
DOTA: 1,4,7,10-tetraazacyclododecane-1,4,7,10-tetraacetate
DTPA: diethylenetriamine-N,N,*N*′,*N*″,*N*″-pentaacetate
ID: injected dose
iTLC: instant thin layer chromatography
LFSE: ligand field stabilization energy
MRI: magnetic resonance imaging
NHE: normal hydrogen electrode
PCTA: 2,2′,2’’-[3,6,9-triaza-1(2,6)-pyridinacyclodecaphane-3,6,9-triyl]triacetate
PET: positron emission tomography
RCY: radiochemical yield
ROI: regions of interest
SUV: standard uptake values
SPE: solid-phase extraction
