# Supplementary material for: Improving the In Vivo Stability of [52Mn]Mn(II) Complexes with 18-Membered Macrocyclic Chelators for PET Imaging
Source: J Med Chem. 2024 Jun 27;67(13):11242–53. doi: 10.1021/acs.jmedchem.4c00812 (PMC11247486; doi:10.1021/acs.jmedchem.4c00812)
Supplement: Supplementary file 1 — jm4c00812_si_001.pdf [file jm4c00812_si_001.pdf]

Supporting Information for:

Improving the *In vivo* Stability of [^{52}Mn]Mn(II) Complexes with 18-Membered Macrocyclic Chelators for PET Imaging

Charlene Harriswangler,[†] James M. Omweri,[‡,§] Shefali Saini,[‡,§] Laura Valencia,[‖] David Esteban-Gómez,[†] Madalina Ranga, [⊥] Nicol Guidolin, [⊥] Zsolt Baranyai,[⊥] Suzanne E. Lapi,[*,‡,§] and Carlos Platas-Iglesias,[*,†]

[†] Universidade da Coruña, Centro Interdisciplinar de Química e Bioloxía (CICA) and Departamento de Química, Facultade de Ciencias, 15071, A Coruña, Galicia, Spain.

[‡] Department of Chemistry, University of Alabama at Birmingham, Birmingham, AL 35205, USA.

[§] Department of Radiology, University of Alabama at Birmingham, Birmingham, AL 35294, USA.

[‖] Departamento de Química Inorgánica, Facultad de Ciencias, Universidade de Vigo, As Lagoas, Marcosende, 36310 Pontevedra, Spain.

[⊥] Bracco Imaging SpA, CRB Trieste, AREA Science Park, ed. Q – S.S. 14 Km 163,5, 34149 Basovizza, TS, Italy.

*E-mail: carlos.platas.iglesias@udc.es
*E-mail: lapi@uab.edu

Summary

Figure S1. Experimental high resolution mass spectrum (ESI^+) of compound Mn-PYAN 4

Figure S2. Experimental high resolution mass spectrum (ESI^+) of compound Mn-CHXPYAN...4

Figure S3. HPLC analysis of Mn-PYAN, retention time 3.17 min.................................5

Figure S4. HPLC analysis of Mn-CHXPYAN.retention time 3.33 min. 5

Figure S5. Plots of the linear dependence of anodic and cathodic peak currents with the square root of the scan rate for Mn-PYAN. 6

Figure S6. Plots of the linear dependence of anodic and cathodic peak currents with the square root of the scan rate for Mn-CHXPYAN. 6

Figure S7. Absorption spectra and absorbance values of the Mn-CHXPYAN- Cu^{2+} reacting system ([MnL]=0.1 mM, [Cu^{2+}]=2.0 mM, [1,4-dimethylpiperazine]=10 mM, pH=3.54, 0.15 M NaCl, 25°C). 7

Figure S8. Radio-TLC chromatograms showing: a) Buffered [^{52}Mn]$MnCl_2$ (R_f = 1.0), b) [^{52}Mn]Mn-CHXPYAN (R_f = 0.39), and c) [^{52}Mn]Mn-PYAN (R_f = 0.35) 7

Figure S9. Radio-TLC chromatograms of the stability study of [^{52}Mn]Mn-PYAN in human serum, mouse serum, and DTPA 8

Figure S10. Radio-TLC chromatograms of the stability study of [^{52}Mn]Mn-CHXPYAN in human serum, mouse serum, and DTPA 8

Figure S11. Radio-TLC chromatograms for the stability study of [^{52}Mn]Mn-CHXPYAN in 10 molar excess of biologically relevant metal ions. 9

Figure S12. Radio-TLC chromatograms for the stability study of [^{52}Mn]Mn-PYAN in 10 molar excess of biologically relevant metal ions. 9

Figure S13. Stability studies of (a) [^{52}Mn]Mn-CHXPYAN and (b) [^{52}Mn]Mn-PYAN in DTPA (5 equivalents), human and mouse. Stability studies of (c) [^{52}Mn]Mn-CHXPYAN and (d) [^{52}Mn]Mn-PYAN in 10 molar excess of 100 μM of Cu^{2+}, Zn^{2+}, Fe^{2+}, and Mg^{2+} over 5 days *(Each data point is n = 3).* The final pH of metal and DTPA challenge samples was ~5.5).. 10

Table S1: Shape analysis of the Mn(II) complexes. A value of 0 indicates that the coordination polyhedron is fully coincident with the reference polyhedron while the maximum value is 100...11

Table S2: HPLC conditions for the purification of the chelators (A = H_2O+0.1% Formic acid, B = CH_3CN+0.1% Formic acid). Flow rate = 4.0 ml/min, samples were dissolved in 1:2 H_2O:CH_3CN. 11

Table S3. Crystal Data and Structure Refinement Details. 11

Table S4: Biodistribution results of [^{52}Mn]Mn-CHXPYAN and [^{52}Mn]Mn-PYAN compared to [^{52}Mn]$MnCl_2$ at 1.5 h postinjection. 12

Table S5: Radioactivity associated with the supernatant and protein pellet upon incubation of [^{52}Mn]Mn-CHXPYAN in mouse serum at 37 ºC. 12

Table S6: Radioactivity associated with the supernatant and protein pellet upon incubation of [^{52}Mn]Mn-CHXPYAN in human serum at 37 ºC. 13

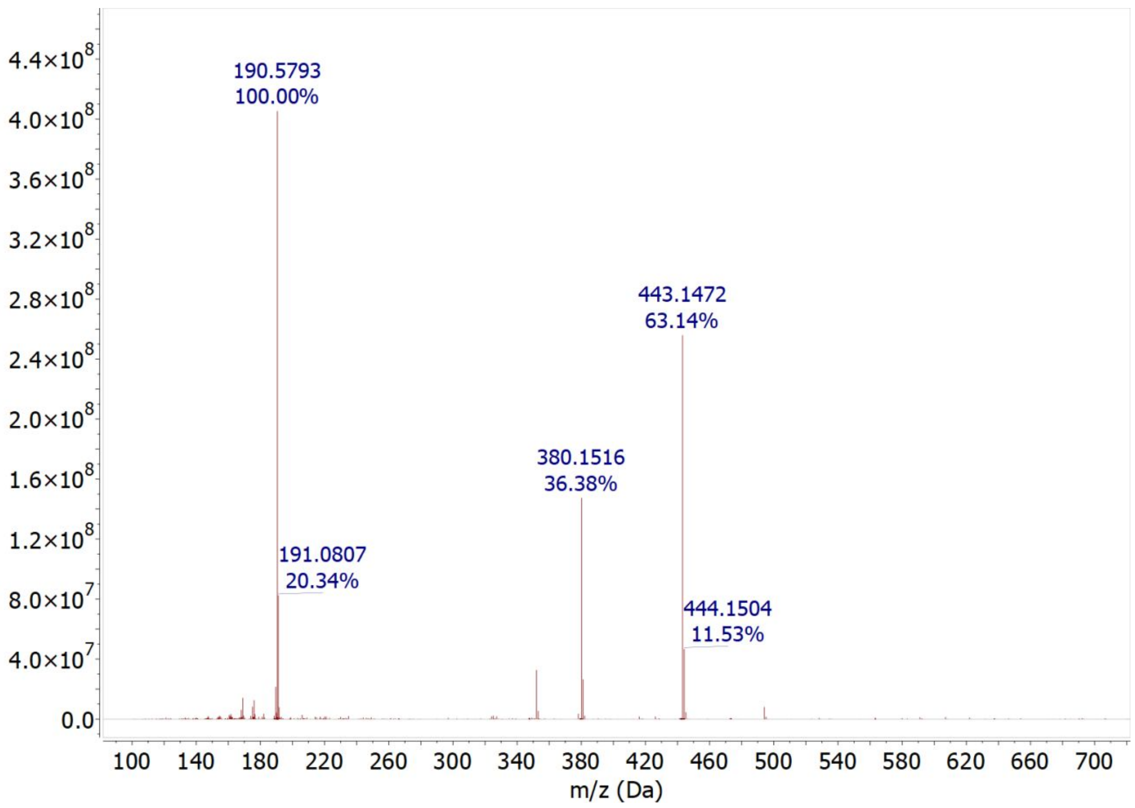

Figure S1. Experimental high resolution mass spectrum (ESI^+) of compound Mn-PYAN

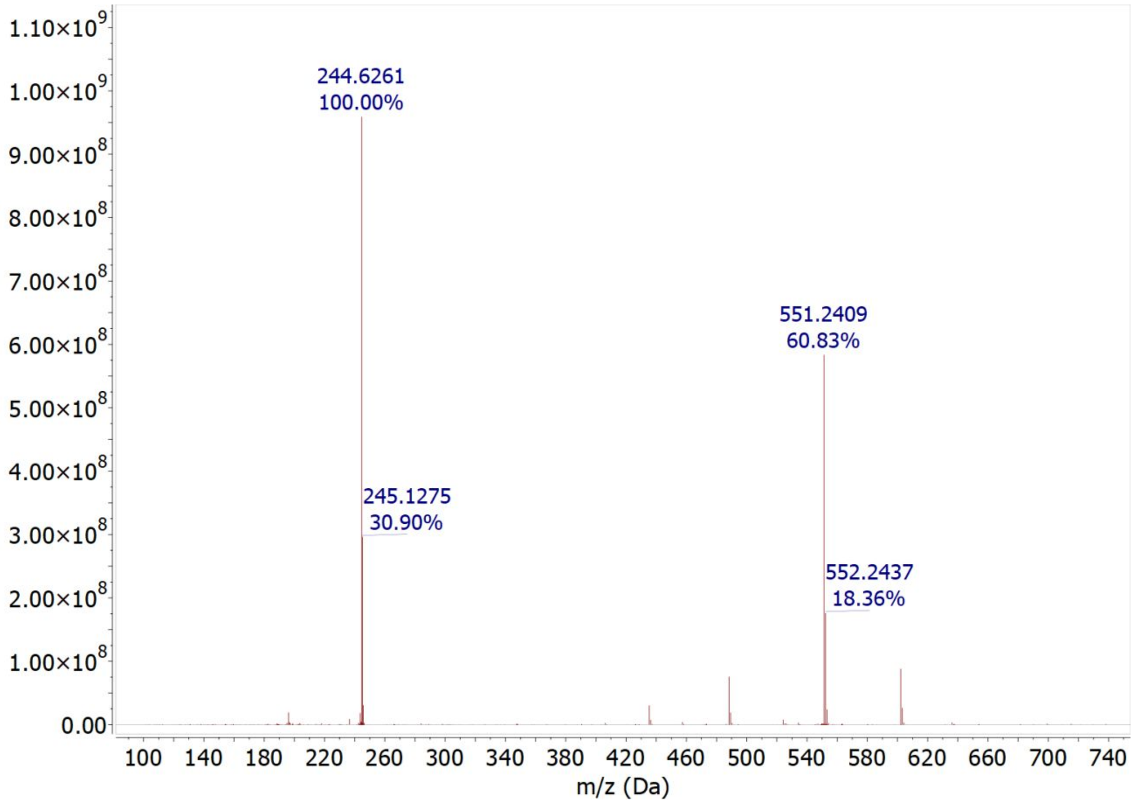

Figure S2. Experimental high resolution mass spectrum (ESI^+) of compound Mn-CHXPYAN.

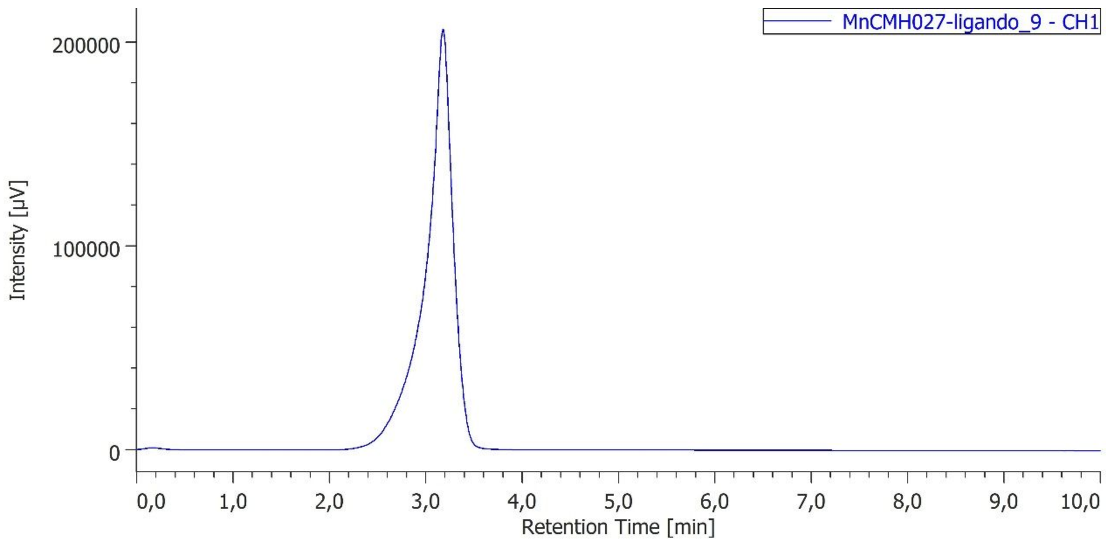

Figure S3. HPLC analysis of Mn-PYAN, retention time 3.17 min.

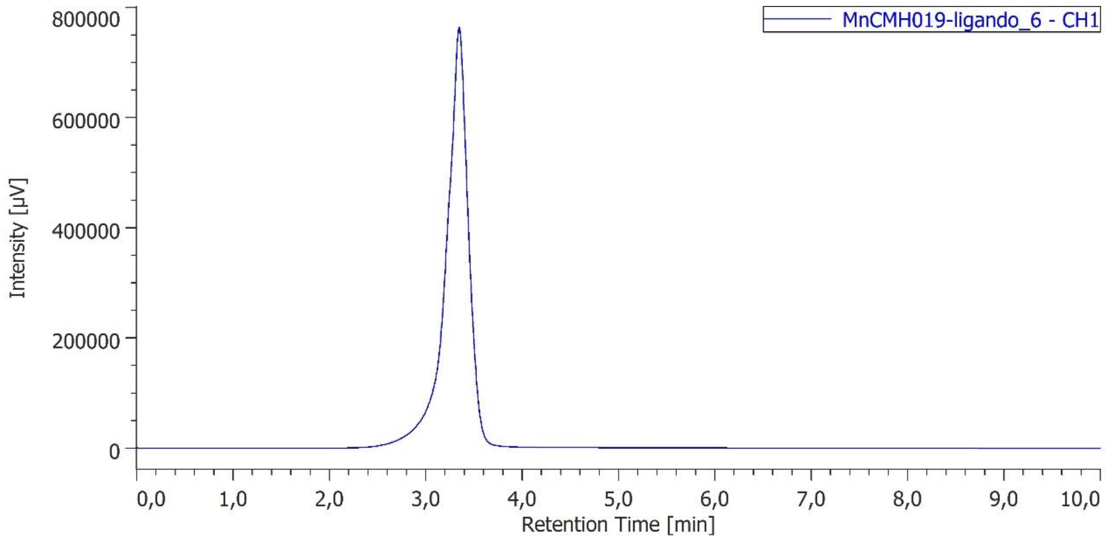

Figure S4. HPLC analysis of Mn-CHXPYAN.retention time 3.33 min.

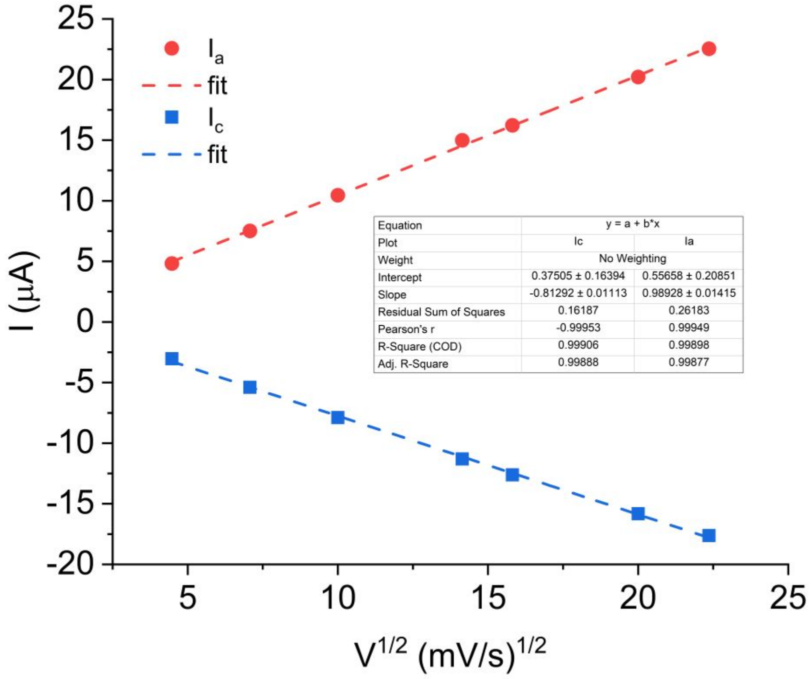

Figure S5. Plots of the linear dependence of anodic and cathodic peak currents with the square root of the scan rate for Mn-PYAN.

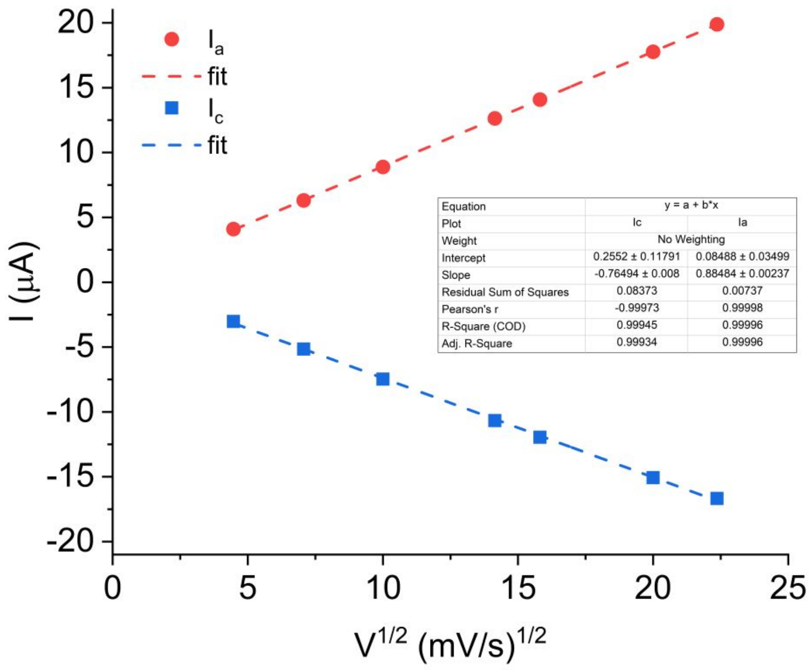

Figure S6. Plots of the linear dependence of anodic and cathodic peak currents with the square root of the scan rate for Mn-CHXPYAN.

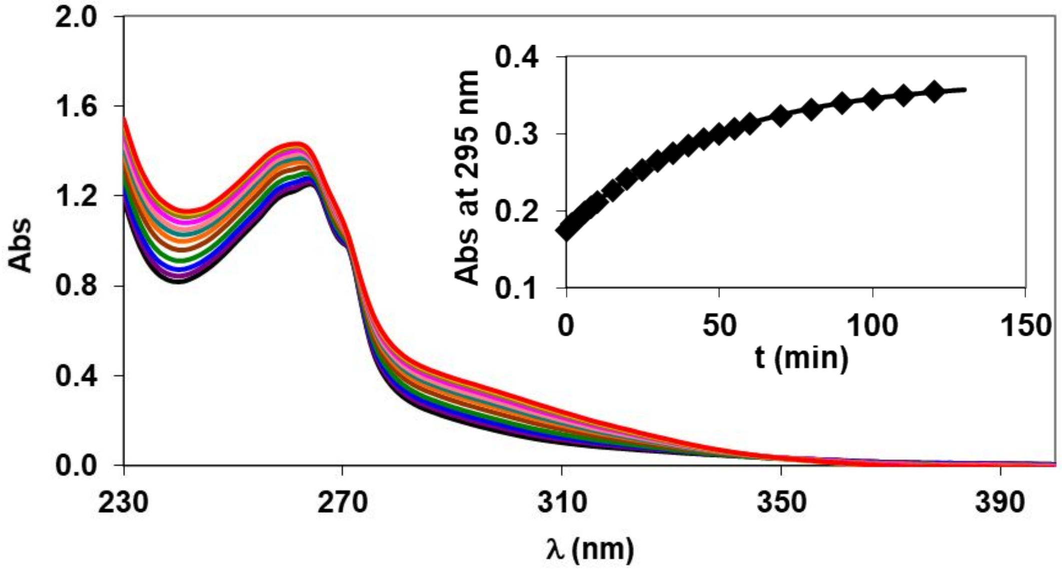

Figure S7. Absorption spectra and absorbance values of the Mn-CHXPYAN- Cu^{2+} reacting system ([MnL]=0.1 mM, [Cu^{2+}]=2.0 mM, [1,4-dimethylpiperazine]=10 mM, pH=3.54, 0.15 M NaCl, 25°C).

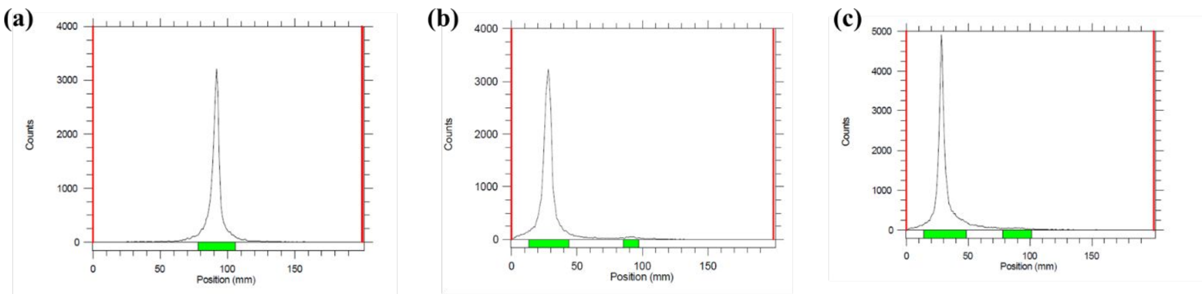

Figure S8. Radio-TLC chromatograms showing: a) Buffered [^{52}Mn]$MnCl_2$ (R_f = 1.0), b) [^{52}Mn]Mn-CHXPYAN (R_f = 0.39), and c) [^{52}Mn]Mn-PYAN (R_f = 0.35)

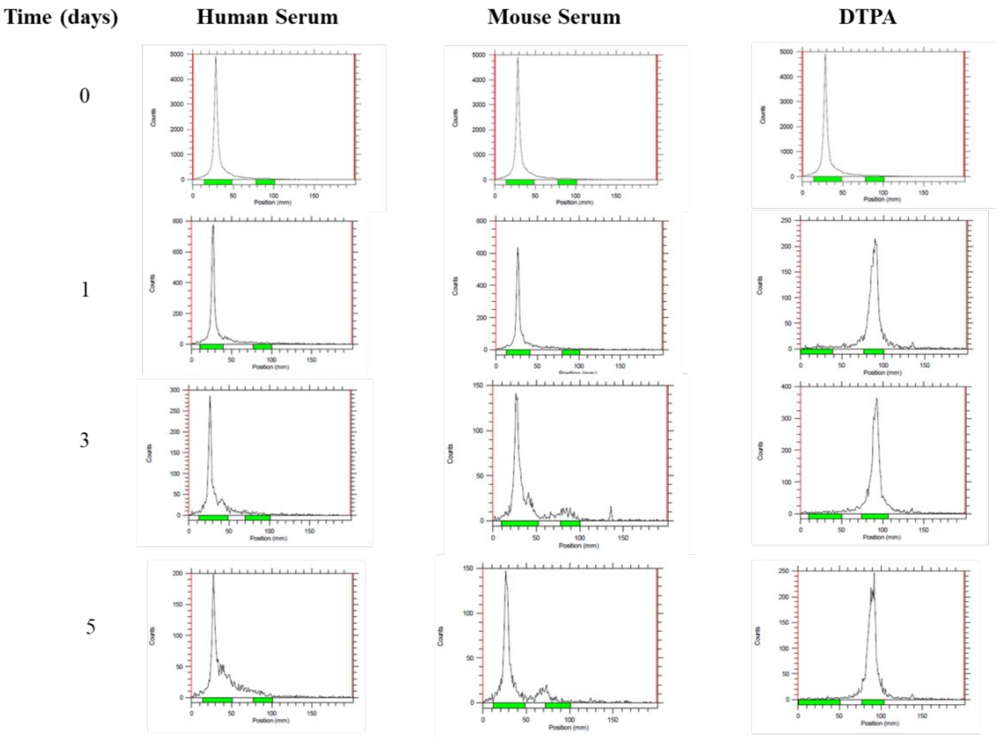

Figure S9. Radio-TLC chromatograms of the stability study of [^{52}Mn]Mn-PYAN in human serum, mouse serum, and DTPA

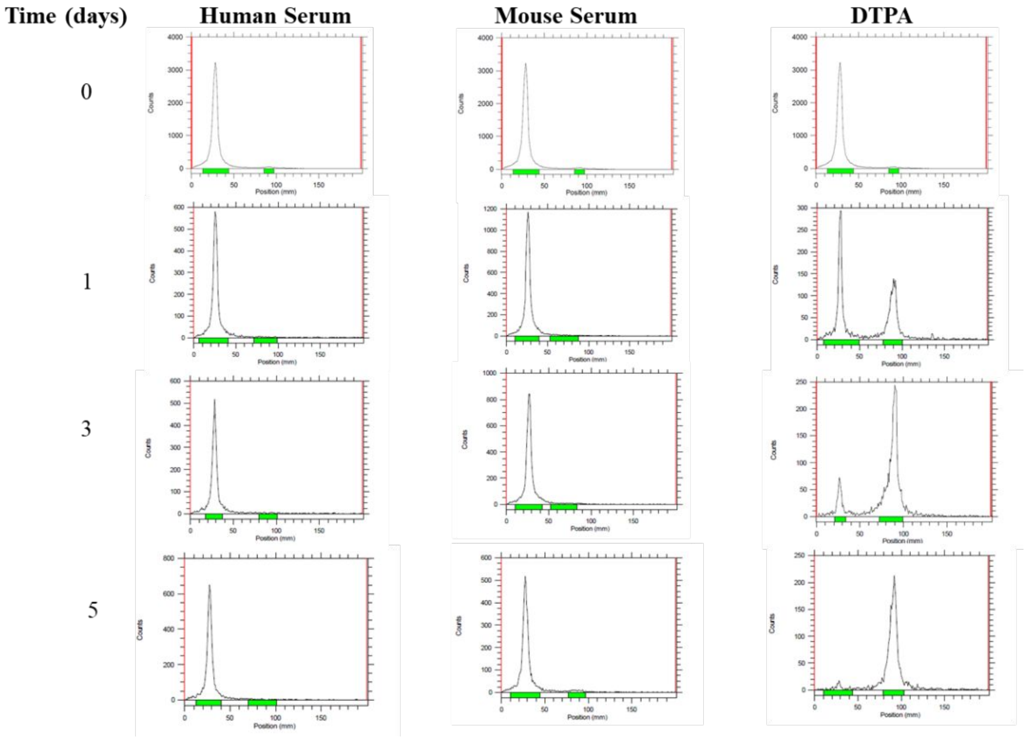

Figure S10. Radio-TLC chromatograms of the stability study of [^{52}Mn]Mn-CHXPYAN in human serum, mouse serum, and DTPA

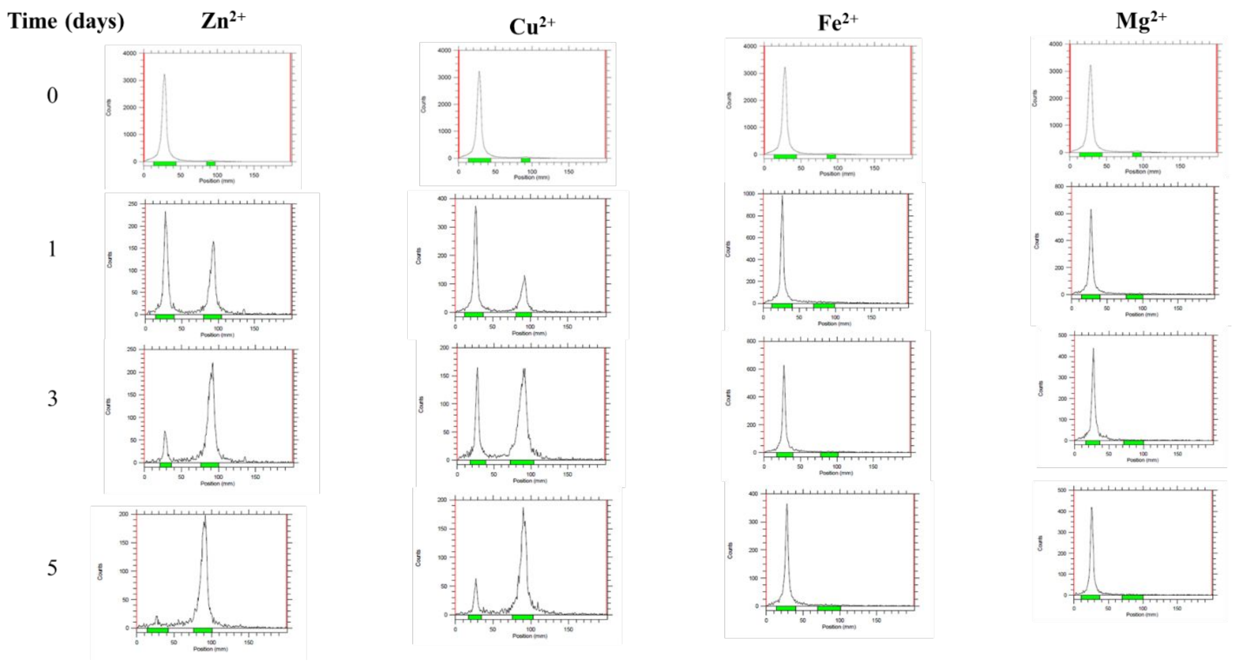

Figure S11. Radio-TLC chromatograms for the stability study of [^{52}Mn]Mn-CHXPYAN in 10 molar excess of biologically relevant metal ions**.**

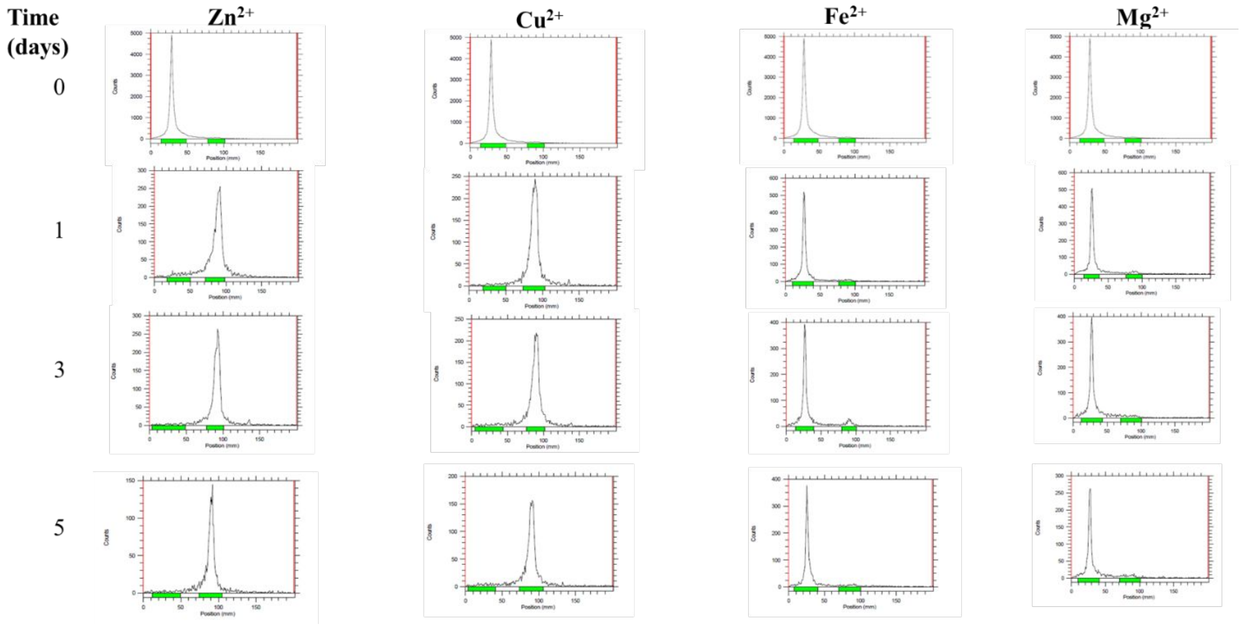

Figure S12. Radio-TLC chromatograms for the stability study of [^{52}Mn]Mn-PYAN in 10 molar excess of biologically relevant metal ions**.**

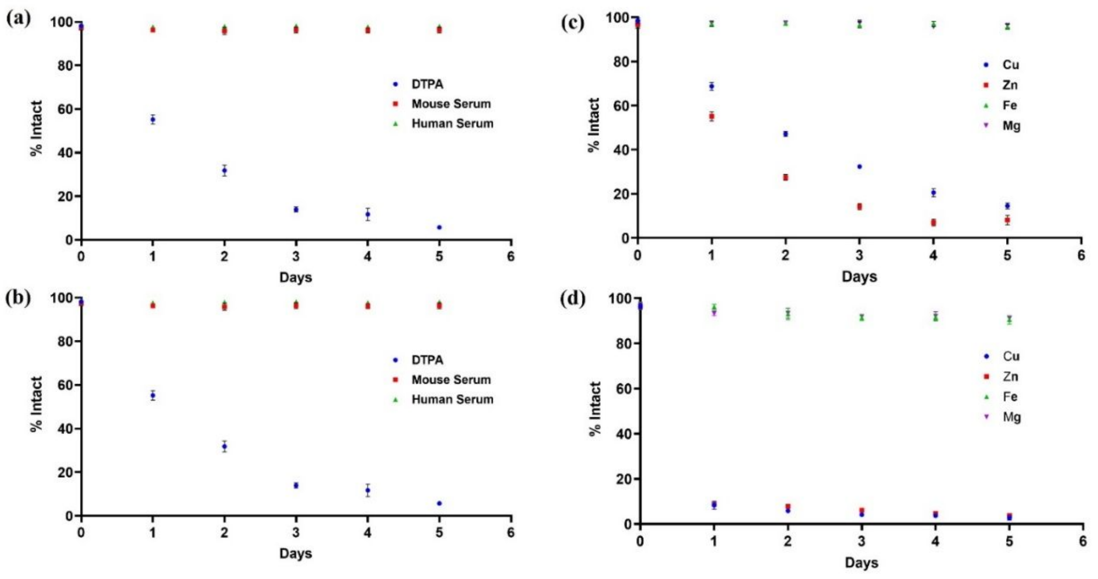

Figure S13. Stability studies of (a) [^{52}Mn]Mn-CHXPYAN and (b) [^{52}Mn]Mn-PYAN in DTPA (5 equivalents), human and mouse. Stability studies of (c) [^{52}Mn]Mn-CHXPYAN and (d) [^{52}Mn]Mn-PYAN in 10 molar excess of 100 µM of Cu^{2+}, Zn^{2+}, Fe^{2+}, and Mg^{2+} over 5 days *(Each data point is n = 3)*. The final pH of metal and DTPA challenge samples was ~5.5)**..**

Table S1: Shape analysis of the Mn(II) complexes. A value of 0 indicates that the coordination polyhedron is fully coincident with the reference polyhedron while the maximum value is 100.

Complex	Hexagon	Pentagonal pyramid	Octahedron	Trigonal prism	Johnson pentagonal pyramid
[Mn(PYAN)]$(PF_6)_{0,5}(NO_3)_{1,5}$	24.270	18.270	7.616	9.109	21.834
[Mn(PYAN)]$(PF_6)_2$	23.120	18.669	7.613	10.891	21.892
[Mn(CHXPYAN)]$(PF_6)_2$	24.792	18.151	6.566	9.868	21.409

Table S2: HPLC conditions for the purification of the chelators (A = H_2O+0.1% Formic acid, B = CH_3CN+0.1% Formic acid). Flow rate = 4.0 ml/min, samples were dissolved in 1:2 H_2O:CH_3CN.

Chelator	Conditions	Retention time (min)
PYAN	Isocratic 95% A (2.5 min)	3.00
CHXPYAN	95 to 5 % A (15 min)<br>Isocratic 5% A (2.5 min)	8.59

Table S3. Crystal Data and Structure Refinement Details.

	**[Mn(PYAN)]$(PF_6)_{0,5}(NO_3)_{1,5}$**	**[Mn(PYAN)]$(PF_6)_2$**	**[Mn(CHXPYAN)]$(PF_6)_2$**
Empirical formula	$C_{18}H_{30}N_{7.5}O_{6.5}F_3P_{0.5}Mn$	$C_{54}H_{80}N_{18}OF_{36}P_6Mn_3$	$C_{26}H_{38}N_6F_{12}P_2Mn$
Molecular weight MW	582.92	2032.00	779.50
Crystal system	Monoclinic	Monoclinic	Monoclinic
Space group	C2/c	P21/c	P21
a/Å	33.6304(17)	9.1503(4)	9.3395(5)
b/Å	8.1363(4)	25.2246(11)	18.6269(8)
c/Å	21.6554(12)	33.9923(17)	18.9772(10)
β/º	122.680(2)	92.381(2)	99.279(2)
Volume ($Å^3$)	4987.5(5)	7839.1(6)	3258.2(3)
Z	8	4	4
ρ_{calc} (g/cm^3)	1.553	1.722	1.589
μ (mm^{-1})	0.636	0.736	0.601
θ range	2.24º - 28.32º	1.97º - 28.37º	2.30º - 28.34º
R_{int}	0.0372	0.0794	0.0538
Measured reflections	73363	194012	116433
Independent reflections / unique (I >2σ (I)	6188 / 5909	19573 / 15471	16149 / 14967
Goodness-of-fit on F^2	1.055	1.126	1.057
R_1	0.0310	0.0636	0.0343
wR_2 (all data)	0.0782	0.1299	0.0883
Larg. diff. peak and hole ($eÅ^{-3}$)	0.76 and -0.66	1.11 and -1.20	0.84 and -0.40

Flack parameter			0.000(7)

Table S4: Biodistribution results of [^{52}Mn]Mn-CHXPYAN and [^{52}Mn]Mn-PYAN compared to [^{52}Mn]$MnCl_2$ at 1.5 h postinjection.

	% ID/g (mean ± SD) *n = 4*		
**Organs**	**[^{52}Mn]Mn-CHXPYAN**	**[^{52}Mn]Mn-PYAN**	**[^{52}Mn]$MnCl_2$**
Blood (μL)	0.14 ± 0.01	0.70 ± 0.20	0.60 ± 0.11
Heart	0.21 ± 0.01	1.85 ± 0.71	12.14 ± 7.31
Lungs	0.26 ± 0.12	1.28 ± 0.51	3.64 ± 2.08
Pancreas	0.20 ± 0.09	2.41 ± 1.60	3.64 ± 2.08
Spleen	0.22 ± 0.18	1.33 ± 0.93	4.83 ± 2.04
Stomach	0.08 ± 0.04	1.29 ± 0.53	4.78 ± 3.02
Liver	1.72 ± 0.93	30.39 ± 11.46	13.00 ± 6.07
Kidney	11.27 ± 1.11	63.34 ± 8.23	38.14 ± 6.06
S. Intestine	2.32 ± 0.83	13.00 ± 2.35	14.23 ± 5.70
L. Intestine	3.45 ± 1.44	2.69 ± 0.82	6.48 ± 1.56
Fat	0.11 ± 0.13	1.03 ± 0.50	1.12 ± 0.53
Skin	0.08 ± 0.04	0.32 ± 0.18	0.31 ± 0.11
Muscle	0.02 ± 0.01	0.18 ± 0.05	0.25 ± 0.13
Femur	0.09 ± 0.04	0.80 ± 0.30	2.42 ± 0.36
Brain	0.03 ± 0.01	0.45 ± 0.09	0.54 ± 0.26

Table S5: Radioactivity associated with the supernatant and protein pellet upon incubation of [^{52}Mn]Mn-CHXPYAN in mouse serum at 37 ºC.

	**CHXPYAN_Mouse serum**			
**Day**	**% activity of supernatant**		**% activity of pellet**	
	Average	SD	Average	SD
1	89.66	2.62	10.33	2.62
2	89.18	1.5	10.82	1.5
3	87.93	1.49	12.07	1.49
4	85.66	1.75	14.33	1.76
5	87.67	2.51	12.14	2.52

Table S6: Radioactivity associated with the supernatant and protein pellet upon incubation of [^{52}Mn]Mn-CHXPYAN in human serum at 37 ºC.

CHXPYAN_Human serum				
**Day**	**% activity of supernatant**		**% activity of pellet**	
	Average	SD	Average	SD
1	86.91	4.53	13.09	4.54
2	87.45	3.03	12.54	3.03
3	85.54	1.32	14.46	1.33
4	89.66	1.75	10.33	1.76
5	90.7	1.63	9.31	1.73
